# Seascape genomics of eastern oyster (*Crassostrea virginica*) along the Atlantic coast of Canada

**DOI:** 10.1111/eva.12741

**Published:** 2018-12-26

**Authors:** Simon Bernatchez, Amanda Xuereb, Martin Laporte, Laura Benestan, Royce Steeves, Mark Laflamme, Louis Bernatchez, Martin A. Mallet

**Affiliations:** ^1^ Institut de Biologie Intégrative et des Systèmes (IBIS) Université Laval Québec Québec Canada; ^2^ Department of Ecology and Evolutionary Biology University of Toronto Toronto Ontario Canada; ^3^ Fisheries and Oceans Canada Moncton New Brunswick Canada; ^4^ L’Étang Ruisseau Bar Ltd. Shippagan New Brunswick Canada

**Keywords:** aquaculture, genomics, genotype by sequencing, oyster, population genetics, RADSeq

## Abstract

Interactions between environmental factors and complex life‐history characteristics of marine organisms produce the genetic diversity and structure observed within species. Our main goal was to test for genetic differentiation among eastern oyster populations from the coastal region of Canadian Maritimes against expected genetic homogeneity caused by historical events, taking into account spatial and environmental (temperature, salinity, turbidity) variation. This was achieved by genotyping 486 individuals originating from 13 locations using RADSeq. A total of 11,321 filtered SNPs were used in a combination of population genomics and environmental association analyses. We revealed significant neutral genetic differentiation (mean *F*_ST_ = 0.009) between sampling locations, and the occurrence of six major genetic clusters within the studied system. Redundancy analyses (RDAs) revealed that spatial and environmental variables explained 3.1% and 4.9% of the neutral genetic variation and 38.6% and 12.2% of the putatively adaptive genetic variation, respectively. These results indicate that these environmental factors play a role in the distribution of both neutral and putatively adaptive genetic diversity in the system. Moreover, polygenic selection was suggested by genotype–environment association analysis and significant correlations between additive polygenic scores and temperature and salinity. We discuss our results in the context of their conservation and management implications for the eastern oyster.

## 1 INTRODUCTION

The population genetic structure of organisms is influenced by complex interactions between evolutionary forces, life‐history characteristics and environmental conditions. For decades, most marine species were assumed to be genetically homogeneous due to their high dispersal potential and to the apparent absence of physical barriers in the oceanic environment (Charrier, Coombs, McQuinn, & Laroche, 2007; Fratini, Schubart, & Ragionieri, 2011; Purcell, Cowen, Hughes, & Williams, 2006; Ward, Woodwark, & Skibinski, 1994): early work using low‐resolution genetic markers (e.g., allozymes, microsatellites) seemed to confirm this (Hauser & Carvalho, 2008). As a result, marine species have been understudied in relation to spatial and environmental (i.e., seascape) factors driving their evolution (Selkoe et al., 2016). Recently, advances in genomic tools, with sufficient resolution to study population genetic processes in marine systems, have resulted in considerable interest in the fast‐growing field of marine and seascape genomics (Gagnaire & Gaggiotti, 2016; Selkoe et al., 2016) and the ability for genomics to enhance management of marine resources (Bernatchez et al., 2017). These studies have improved our understanding of how genomic variation in marine and coastal ecosystems is spatially distributed, and how these patterns are influenced by environmental conditions (Benestan et al., 2015; DiBattista et al., 2017; Diopere et al., 2017; Lal, Southgate, Jerry, Bosserelle, & Zenger, 2017; Metivier, Kim, & Addison, 2017; Sandoval‐Castillo, Robinson, Hart, Strain, & Beheregaray, 2018; Van Wyngaarden et al., 2018; Xuereb, Benestan, et al., 2018; Xuereb, Kimber, Curtis, Bernatchez, & Fortin, 2018). While marine species are typically characterized by low absolute levels of genetic differentiation relative to their terrestrial counterparts, these studies have revealed complex interactions between spatial and environmental processes, mediated by a species’ particular life‐history traits, operating at a different scale than in familiar terrestrial systems.

While many marine species have important ecological and economic roles, management decisions are often made without critical population genetic information (Bernatchez et al., 2017). This is particularly true for invertebrate species, which in general have received much less attention than marine vertebrates. Bivalves in particular occupy important ecological and economic roles in coastal ecosystems throughout the world, and have been the object of intense anthropological influence (Dumbauld, Ruesink, & Rumrill, 2009; Martinelli, Soto, González, & Rivadeneira, 2017; National Research Council, 2010; Shumway, 2011).

Oysters are the most economically important groups of bivalves (FAO, 2018), as well as being valued for the important ecosystem services they provide (zu Ermgassen, 2013). In North America, the eastern oyster (*Crassostrea virginica* Gmelin) has historically been the most important bivalve species. This species occupies the shallow waters of bays, lagoons and estuaries along the east coast of North America (Jackson, 2001; Policy & Economics Branch, Gulf Region, 2003). It has a planktonic larval stage of approximately three to four weeks (Booth & Sephton, 1993; Policy & Economics Branch, Gulf Region, 2003; Vercaemer, St‐Onge, Spence, Gould, & McIsaac, 2010), during which the larvae can perform active vertical movement and thus bias their drift horizontally using horizontal and tidal currents (Abbe, 1986; Andrews, 1983; Mann, 1988; Seliger, Boggs, Rivkin, Biggley, & Aspden, 1982). After this period, the larvae settle themselves on hard surfaces by ejecting an adhesive, become “spat” (juvenile) and remain sessile for the rest of their lives (Eastern oyster Biological Review Team, 2007; Policy & Economics Branch, Gulf Region, 2003). *Crassostrea virginica* is naturally distributed from the Gulf of Mexico to New Brunswick, Canada, where it historically formed reefs comprised of millions of individuals (Wilberg, Livings, Barkman, Morris, & Robinson, 2011), providing physical structure for a range of marine species and important filtration services (Buroker, 1983; National Oceanic and Atmospheric Administration (NOAA) 2018a). Many of these large oyster reefs have been disrupted by overfishing in the 19th‐20th centuries, and contemporary oyster populations are thought to be at less than 1% of their historical abundance (Mackenzie, 2007; Wilberg et al., 2011). The eastern oyster is still the object of an important wild‐capture fishery and aquaculture industry throughout its range (FAO, 1992), and several major restoration projects are currently underway in the United States (Hudson River Foundation, 2018; NOAA, 2018b; Oyster Restoration Workgroup, 2018; Virginia Department of Environment Quality, 2018).

In Canada, oysters have been an important resource for indigenous peoples and colonists (Lavoie, 1978). As in the rest of its range, Canadian oyster beds were subject to intense exploitation, to which they are especially vulnerable due to their slower growth and maturation at this northern range limit (Comeau, Mayrand, & Mallet, 2012). In an effort to replenish the fisheries, oyster spat capture and translocation has been long‐standing features of the fishery practices which are thought to have contributed to the introduction and spread of a major epizootic event, now known as Malpeque disease.

In 1915, mass mortality events were noted in Malpeque Bay, Prince Edward Island (PEI), Canada, and are thought to have been caused by transfer of oyster spat from New England in 1913–1914, in an effort to rebuild overharvested stocks (McGladdery & Zurbrigg, 2006; McGladerry & Bower, 1999; Medcof, 1961). Despite regulations prohibiting the transfer of oysters from Malpeque Bay after mortality was observed, the disease spread to other PEI bays, with characteristic mass mortalities of >90% across all age classes. Eventually, the Malpeque stock appeared to acquire resistance to Malpeque disease and disease‐resistant broodstock were transferred to other bays in an effort to accelerate the recovery of oyster populations (McGladdery & Zurbrigg, 2006). By the 1950–1960s, Malpeque disease appeared in New Brunswick (NB) and Nova Scotia (NS), presumably from prohibited transfers of asymptomatic disease carriers from PEI, causing the same widespread mortalities and decimating oyster beds.

The recovery strategy was to replace susceptible oysters from NB and NS with PEI disease‐resistant oysters through mass transplantation of adult oysters and seed (Drinnan, 1967; Drinnan & England, 1965; Drinnan & Medcof, 1961; Found & Logie, 1957; McGladdery & Zurbrigg, 2006). The strategy appeared to be successful in re‐establishing oyster populations, and subsequently, all oyster populations that were subject to these massive transplantations were assumed to be genetically homogeneous (Vercaemer et al., 2010). In addition to these massive historical transfers, spat collection and translocation at all geographic scales (within and between bays, regions and provinces) have been a continuing practice among oyster fishers and aquaculturists (Policy & Economics Branch, Gulf Region, 2003), which is expected to continue to exert a homogenizing influence.

Despite these large historic disturbances, oyster populations in the Maritimes may be genetically differentiated. In the northern part of their range, eastern oysters are found in semi‐closed environment like estuaries, lagoons and bays, and oyster populations from different bays are not contiguous. Coastal habitats in the southern Gulf of Saint Lawrence are considerably fragmented and diversified ecologically (Dutil et al., 2012). Accordingly, selective forces could be large enough to maintain differentiation in the face of gene flow. Additionally, sweepstakes reproductive success (SRS), which refers to a large variance in individual reproductive success (Cushing, 1990; Waples, 1998), has been documented in several shellfish species (including oysters) and could imply an important effect of genetic drift (Hedgecock & Pudovkin, 2011). Yet, a low‐resolution microsatellite study (Vercaemer et al., 2010) found little evidence for differentiation between oyster populations in the Gulf of Saint Lawrence. On the other hand, empirical studies have found population‐of‐origin effects on oyster phenotype for these same populations (Mallet & Haley, 1983; Newkirk, 1978).

Given the increasing importance of eastern oysters as an aquaculture species (Atlantic Canada Opportunities Agency, 2012; Government of New Brunswick, 2018) and the availability of modern, high‐resolution genomic methods, it is important to revisit the population genetic portrait of eastern oyster populations in the Maritime provinces of Canada. We used wild oysters collected from PEI, NB and NS to test the prediction of structured populations of the eastern oyster against that of homogenized genetic structure. We also investigated the influence of spatial and environmental variation on observed patterns of genetic differentiation. This is a critical preliminary step towards improving oyster management and production in Atlantic Canada.

## 2 MATERIALS AND METHODS

### 2.1 Sampling

**Figure 1:**
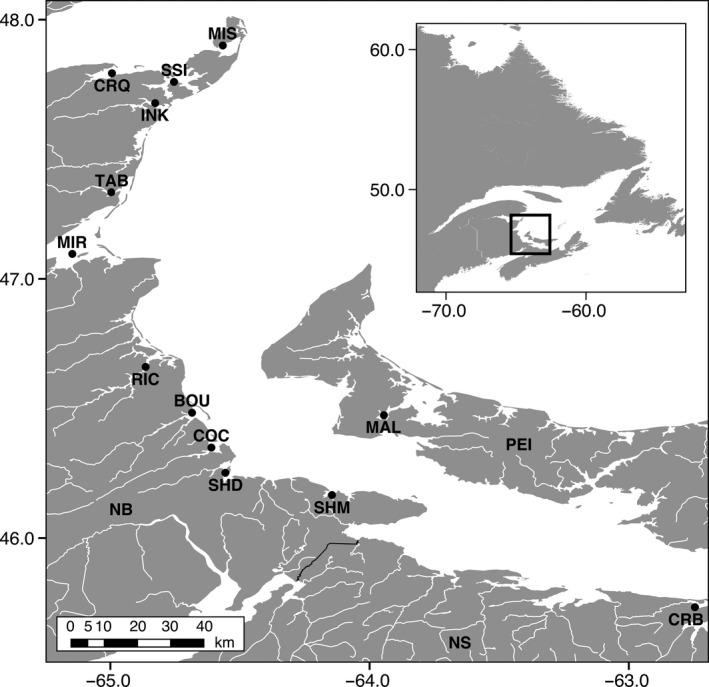
Map of sampling sites within New Brunswick (NB), Nova Scotia (NS) and Prince Edward Island (PEI). BOU: Bouctouche, COC: Cocagne, CRB: Caribou, CRQ: Caraquet, INK: Inkerman, MAL: Malpeque, MIR: Miramichi, MIS: Miscou, RIC: Richibucto, SHD: Shediac, SHM: Shemogue, SSI: Saint‐Simon, TAB: Tabusintac

Oysters were collected in the summer of 2014 by a combination of hand‐picking, snorkelling and dredging in 13 bays, with 11 being situated along the eastern coast of NB, one in NS and one in PEI (Canada) (Figure 1 and Table 1). For NB populations, oysters were sampled from at least five different sites within each bay, and we selected mature oysters (>3‐inches) where possible. For PEI and NS populations, we used archived tissue samples from the Department of Fisheries and Oceans Canada (DFO). We attempted to collect oysters on natural reefs/beds and avoided aquaculture leases to minimize the chance of collecting aquaculture oysters that may have been translocated from other bays. A total of 501 wild oysters were used for this study. Tissues were sampled from the adductor muscle and preserved in 95% ethanol until DNA extraction.

**Table 1:** Location of sampling sites with corresponding number of samples successfully genotyped (*n*). Geographic coordinates are averaged from the subsampling sites

Sites	Abbreviation	Latitude	Longitude	Location	*n*
Caraquet	CRQ	47.7933	−64.9938	NB	40
Miscou	MIS	47.9017	−64.5666	NB	39
Saint‐Simon	SSI	47.7599	−64.7544	NB	30
Inkerman	INK	47.6781	−64.8270	NB	38
Tabusintac	TAB	47.3342	−64.9965	NB	40
Miramichi	MIR	47.0963	−65.1467	NB	37
Richibucto	RIC	46.6611	−64.8646	NB	39
Bouctouche	BOU	46.4834	−64.6849	NB	40
Cocagne	COC	46.3489	−64.6231	NB	38
Shediac	SHD	46.2517	−64.5565	NB	39
Shemogue	SHM	46.1661	−64.1450	NB	38
Caribou	CRB	45.7332	−62.7449	NS	33
Malpeque	MAL	46.4742	−63.9447	PEI	35

Note

NB: New Brunswick; NS: Nova Scotia; PEI: Prince Edward Island.

### 2.2 Library preparation and sequencing

Genomic DNA was extracted using a modified low‐salt CTAB extraction protocol (Arseneau, Steeves, & Laflamme, 2017); DNA quality and quantity were assessed using a NanoDrop 2000 spectrophotometer and agarose gel electrophoresis. RADSeq library preparation was conducted following a slightly modified version of the Peterson, Weber, Kay, Fisher, and Hoekstra (2012) RADSeq protocol employing two restriction enzymes (*NsiI* and *MspI*) (Supporting Information Appendix S1). Paired‐end 100‐bp sequencing was conducted on Illumina HiSeq 2000 platform at the Genome Quebec Innovation Center (McGill University, Montréal, Canada).

### 2.3 Bioinformatics and genotyping

Read quality was evaluated using FastQC v0.11.5 (Andrews, 2010). We used the STACKS module *process_radtags* v1.40 (Catchen, Amores, Hohenlohe, Cresko, & Postlethwait, 2011; Catchen, Hohenlohe, Bassham, Amores, & Cresko, 2013) to demultiplex samples, remove inline barcodes, truncate reads to a common length and apply quality filtering parameters. The first four bases of all demultiplexed sequences were trimmed using TRIMMOMATIC v0.32 (Bolger, Lohse, & Usadel, 2014) to remove low‐quality and ambiguous bases, resulting in a final read length of 90 bp. Then, STACKS v1.44 was used for the identification of the loci and the calling of genotypes. Reads were aligned against the eastern oyster genome (NCBI Bioprojects: PRJNA379157 and PRJNA376014, accession numbers: NC_007175.2 and NC_035780.1 – NC_035789.1) using *pstacks*. We used a minimum depth of coverage of two (*m* = 2) to create a stack, the SNP model with an alpha of 0.05 and a minimum percentage of alignment of 85% to keep a read. A catalog of putative loci was created (*cstacks*) based on alignment position and allowing one mismatch between sample loci. The *populations* module was then used to call genotypes, applying several filtering steps to ensure quality of the data. First, a SNP had to be genotyped in at least 80% of the individuals in at least 12 of the 13 sampling sites. This criterion was used to minimize missing data, while keeping a substantial number of informative SNPs in our system. Second, in order to have sufficient power to call heterozygote genotypes, a SNP had to have a minimum read depth of 5. Third, a SNP had to have a global minor allele frequency (MAF) ≥0.01 or a local MAF ≥0.05. This filtering step is used to exclude putative sequencing errors and keep SNPs that are the most informative in our system. Only SNPs with a heterozygosity ≤0.6 for at least 12 of the 13 sampling sites were kept in order to avoid assembling homeologs as single loci. Individuals with more than 12% of missing data were excluded from the pipeline. Finally, we removed SNPs originating from the mitochondrial genome and SNPs sharing the same position within a single chromosome (duplicated SNPs resulting from sequencing and pipeline artefacts).

We generated two data sets: (a) a haplotype data set for analyses of genetic diversity and (b) a SNP data set for analyses of population genetic structure. For the haplotype data set, we excluded loci for which there were more than two haplotypes in five individuals or more (we discarded individuals with more than two haplotypes) and a maximum of 20% missing haplotypic data. We added consensus sequences with a maximum of 20% missing data to this data set. For the SNP data set, we kept only the SNPs with the maximum MAF at each locus. Details of the number of loci and SNPs remaining after each filtering steps are presented in Supporting information Table S1. Filtering and file conversions were performed using R v3.4.3 (R Development Core Team 2017) and PYTHON software (http://www.python.org/) scripts, PGDSPIDER v.2.1.1.3 (Lischer & Excoffier, 2012), VCFTOOLS software v0.1.15 (Danecek et al., 2011), PLINK software v1.90b5 (Purcell et al., 2007) and custom scripts (https://github.com/enormandeau). Patterns of identity by missingness (IBM) were examined using a multidimensional scaling analysis implemented in PLINK to evaluate clustering bias that could be related to missing data shared between sequencing lanes or sampling sites.

### 2.4 Detecting SNPs putatively under selection and defining data sets

Loci under selection are expected to behave differently from neutral loci in terms of population‐related patterns of diversification and are thus inappropriate to infer population demographic history (Beaumont & Nichols, 1996; Holderegger, Kamm, & Gugerli, 2006; Luikart, England, Tallmon, Jordan, & Taberlet, 2003). We identified SNPs as being putatively neutral or under selection using three differentiation‐based (*F*_ST_) outlier detection methods, which use different underlying models: (a) BAYESCAN v2.1 (Foll & Gaggiotti, 2008) with prior model of 10 000, following the recommendations of Lotterhos and Whitlock (2015), 10,000 iterations, a burn‐in of 200,000 steps and a *Q*‐value threshold of 0.05; (b) ARLEQUIN v3.5 (Excoffier & Lischer, 2010) with 200,000 simulations and 1,000 demes; and (c) OUTFLANK v0.2 (Whitlock & Lotterhos, 2015) with default options (LeftTrimFraction = 0.05, RightTrimFraction = 0.05, Hmin = 0.1, 13) and a Q‐threshold of 0.05. With ARLEQUIN, we used a false discovery rate (FDR) correction method to minimize the number of false‐positive (type I errors) outlier loci and we set a corrected *P* threshold of 0.05 to consider a locus as an outlier. The results of the three analyses were used to define a putatively neutral SNP data set, as well as a data set of SNPs putatively under divergent selection based on outliers detected using all three methods. Finally, as some analyses assume that genetic markers are not strongly linked, we tested the SNP data set for linkage disequilibrium (LD) (between each pair of SNPs within single chromosomes, *n* = 10) using VCFTOOLS. For each pairwise comparison with an associated *R* ^2^ > 0.8, we randomly removed one linked SNP until no *R* ^2^ value ≥ 0.8 was observed.

### 2.5 Missing data imputation

As some analyses cannot handle missing data, we performed an imputation of missing genotypes (4.7% missing genotypes overall), using the default options of the “on‐the‐fly” Random Forest algorithm (Ishwaran & Kogalur, 2015; Ishwaran, Kogalur, Blackstone, & Lauer, 2008), implemented in the R package RADIATOR v0.0.11 (Gosselin, 2018a). This approach is an efficient machine‐learning method known for its excellent prediction performance and capacity to address interactions and nonlinearity, in addition to avoiding overfitting and measures variable importance for variable selection (Breiman, 2001; Tang & Ishwaran, 2017). This was achieved by first evaluating overall genetic differentiation using the putatively neutral SNPs based on estimates of *F*_ST_ (Weir & Cockerham, 1984) between sampling sites using the R package ASSIGNER v0.5.0 (Gosselin, Anderson, & Bradbury, 2017), with 95% confidence intervals (5,000 bootstraps). The results from this analysis were used to subsequently infer groups required for missing data imputation. Pairwise comparisons between sampling sites with 95% confidence intervals (CIs) including zero were defined as a single group for imputation.

### 2.6 Genetic diversity

The haplotype data set and the R package STACKR v2.0.4 (Gosselin, 2018b) were used to calculate number of monomorphic and polymorphic loci for each sampling site, in addition to nucleotide diversity (*P*_i_, calculated individually) and the number of consensus reads (i.e., reads with no polymorphism throughout the whole data set). The average *P*_i_ values were compared between sampling sites using a *t* test in *R*.

The SNP data set was used to calculate observed (*H*_o_) and expected (*H*_e_) heterozygosity using GENODIVE 2.0b27 (Meirmans & Tienderen, 2004), as well as deviations from the Hardy–Weinberg equilibrium (HWE) with *F*_IS_ inbreeding coefficient (10,000 permutations) using the R package HIERFSTAT (Goudet, 2005; Goudet & Jombart, 2015). These statistics were evaluated for each sampling site using the full set of SNPs and were compared between the nonimputed and imputed data sets.

### 2.7 Genetic differentiation, population structure and population assignment

All analyses related to neutral population genetic structure were performed using the imputed data set of putatively neutral SNPs and excluding SNPs with strong LD (i.e., *R* ^2^ value ≥ 0.8). Differentiation between sampling sites was first evaluated using pairwise estimates of *F*_ST _(Weir & Cockerham, 1984) through the R package ASSIGNER and a FDR adjustment. We used TREEFIT v1.2 (Kalinowski, 2009) to generate a bootstrapped UPGMA dendrogram of *F*_ST_ distances, visualized using FIGTREE v1.4.3 (http://tree.bio.ed.ac.uk/software/figtree/).

Isolation by distance (IBD) was evaluated with a Mantel test of all pairwise *F*_ST_ comparisons as a function of geographic distances between sampling sites using ADEGENET (Jombart, Devillard, & Balloux, 2010). Geographic distances were estimated by using the shortest marine distance between the average sampling site coordinates of each bay. ADEGENET was also used to assess population clustering with a discriminant analysis of principal components (DAPC). We performed DAPC using the optimal number of clusters as determined based on the Bayesian information criterion (BIC) (Fraley & Raftery, 1998; Jombart et al., 2010; Lee, Abdool, & Huang, 2009). A second DAPC was also conducted using the sampling sites as a prior (*K* = 13). The optimal α‐score was used to choose the optimal number of principal components (*n* = 45), and we used all discriminant functions (*n* = 12) for both analyses.

A hierarchical analysis of molecular variance (AMOVA) was performed using GENODIVE. Sampling sites were first grouped according to pairwise *F*_ST_ values (pairs of sites with *F*_ST_ < 0.001 were considered as a single population). A second grouping (hereafter “clusters”) was based on the groups observed using the combined results of the dendrogram and DAPC approaches (see Results).

Finally, an individual assignment test was conducted using the approach developed by Paetkau, Calvert, Stirling, and Strobeck (1995) and implemented in GENODIVE. To avoid the problem of high‐grading bias (see Anderson, 2010; Waples, 2010), we used all markers from the SNP data set (one SNP per locus, imputed data set) as recommended by Benestan, Gosselin et al. (2016). The null distribution of likelihood values was generated using a Monte Carlo test (10,000 permutations; Cornuet, Piry, Luikart, Estoup, & Solignac, 1999). As all possible source populations may have not been sampled, we used the home likelihood criterion (*L*_H_) to detect putative migrants, and a threshold alpha value of 0.002 was used to determine whether individuals were tagged as migrants (see Berry, Tocher, & Sarre, 2004). We performed the assignment analysis by sampling sites and then by clusters. In order to assess the influence of unbalanced sample sizes, for each cluster, we used randomly chosen individuals to form equal sample sizes (*n* = 33, smallest sample size).

### 2.8 Association between spatial structure, environmental factors and genetic variation

We used a spatial eigenfunction approach to evaluate spatial structure between sampling sites based on distance‐based Moran's eigenvector map (dbMEMs). With this approach, physical distances are decomposed into a new set of independent spatial variables (dbMEMs) that can be used as explanatory variables in subsequent analyses (Borcard & Legendre, 2002; Borcard, Legendre, Avois‐Jacquet, & Tuomisto, 2004; Buttigieg & Ramette, 2014; Dray, Legendre, & Peres‐Neto, 2006). We used the same previously described marine distances to generate a distance matrix between all pairs of sampling sites. The *pcnm* function in the R package VEGAN (Oksanen et al., 2018) was used to generate the dbMEMs.

Environmental variables used for analyses were retrieved from a database made available by Fisheries and Oceans Canada (DFO; Dutil et al., 2012). The data set describes the coastal and epipelagic (0–30 m) habitats of the estuary and Gulf of Saint Lawrence in a grid divided into 6.25 km cells. Of the available variables, we chose the ones that we considered the most likely to be important for oysters (eight temperature, three salinity and three turbidity variables). We considered surface sea temperature only, as the available data were more detailed and it tends to be correlated with bottom temperatures in the studied region (Brickman & Drozdowski, 2012; Drinkwater & Gilbert, 2004; Dutil et al., 2012). Correlation between environmental variables was evaluated using the Pearson correlation coefficient. When two variables were highly correlated (|*r*| ≥ 0.7), only one variable was retained. The resulting set of variables included seven variables related to temperature (mean surface temperature, minimum surface temperature, number of weeks with temperature between 2 and 6°C, 6 and 10°C, 10 and 14°C, 14 and 18°C, and above 18°C), two to turbidity (maximum monthly turbidity and minimum monthly turbidity) and one to salinity (mean surface salinity). Missing values (0.4%) for environmental data were replaced with the median using the function *na.roughfix* in the R package RANDOMFOREST (Liaw & Wiener, 2002).

We evaluated the influence of spatial distribution of environmental factors on putatively neutral and adaptive genetic variation throughout the studied system using redundancy analyses (RDAs) (Buttigieg & Ramette, 2014; Legendre & Legendre, 2012). First, we performed two separate RDAs using the spatial (i.e., dbMEM) and environmental explanatory variables and (a) the 8,246 neutral SNPs and (b) the six loci putatively under directional selection (see Results) as the multivariate response variables. We used an analysis of variance (ANOVA) with 1,000 permutations to assess the correlation between genotype and each spatial/environmental variable, one variable at a time. Only variables with a *p*‐value ≤ 0.1 were retained for subsequent analyses. We then used the variance inflation factor (VIF; *vif.cca* function implemented in the R package VEGAN) to evaluate multicollinearity of all retained variables (Hair, Anderson, Tatham, & Black, 1995; James, Witten, Hastie, & Tibshirani, 2017; Zuur, Ieno, & Elphick, 2010) and excluded variables with a VIF ≥ 10 (Hair et al., 1995). Then, we used the *ordistep* function in the R Package VEGAN to select the most important explanatory variables among those retained. This is an automated model building approach that can perform stepwise model selection of constrained ordination methods using permutation tests and associated *p*‐values. The function was thus run on the RDA performed with all previously chosen explanatory variables (i.e., spatial and environmental variables). The global significance of the final RDA was assessed using an ANOVA, and marginal ANOVAs (1,000 permutations) were performed to assess the contribution of each explanatory variable. Partial RDAs were subsequently conducted to assess the proportion of genetic variation explained by environmental factors after controlling for the effect of spatial variables and vice versa. All RDAs were performed using the function *rda* in the R package VEGAN.

We also performed a RDA as a multilocus genotype–environment association method to detect loci putatively under selection based on correlations with environmental variables. This approach can detect (even weak) multilocus signatures of selection for multiple environmental predictors, especially compared to differentiation‐based outlier detection methods (Forester, Lasky, Wagner, & Urban, 2018; Rellstab, Gugerli, Eckert, Hancock, & Holderegger, 2015). We used genotypes for all SNPs (one SNP per locus) and the same environmental variables as in the previous RDAs. The significance (alpha ≤ 0.05) of the global RDA and significance of each RDA axis were assessed using an ANOVA with 1,000 permutations, as above. Outlier SNPs were defined using the distribution of their loadings on each significant RDA axis, where SNPs showing a loading located in the tail of the distribution are more likely to be under selection. We used a cut‐off of ±3 *SD* from the mean loading of each axis to identify candidate SNPs, as suggested by Forester et al. (2018) to minimize false‐positive and false‐negative results. Thereafter, the correlation between each candidate SNP and the environmental variables was evaluated using the Pearson correlation coefficient.

As a final step for the multilocus approach, we calculated a polygenic score to assess the individual cumulative adaptive genetic variation associated with each environmental variable tested (Babin, Gagnaire, Pavey, & Bernatchez, 2017; Gagnaire & Gaggiotti, 2016). For each individual, we used the genotypes (0, 1, 2) to generate a score for each outlier SNP, for which we evaluated the relationship with their correlated environmental variable. If the slope of the relationship was negative, scores were inverted (2, 1, 0) to obtain a positive relationship. Then, at the individual level, we summed the score of each SNP correlated with a particular variable, giving an individual polygenic score for each variable independently. Finally, we tested whether a linear or quadratic model better fit the relationship between the polygenic score and the associated environmental variable according to the lowest Akaike information criterion (AIC) value.

### 2.9 Gene ontology

To gain insight into possible targets of selection, we performed a gene ontology (GO) annotation of SNPs with nonsynonymous mutations. First, as we used only a single SNP for each locus, we extracted all SNPs having passed our quality filters located on loci for which a SNP has been identified as being putatively under selection by at least one method (BAYESCAN, ARLEQUIN, OutFLANK, RDA). The flanking regions (100 bp) of these SNPs were extracted from the eastern oyster reference genome that we previously used for the bioinformatic pipeline and BLASTed (minimum of 80% of similarity on 30 amino acids) on the protein sequences of *C. virginica. *For variants that resulted in the same protein result, we evaluated whether the amino acid sequence was the same or not. If amino acid sequences were different, we conducted a search on the SWISS‐PROT database (Bairoch & Apweiler, 2000) using the protein name.

## 3 RESULTS

### 3.1 Bioinformatics and genotyping

Sequencing of libraries resulted in a total number of 1,514,266,246 raw reads, and the median number of filtered reads obtained per individual was 2,991,344. After removing individuals with low‐quality data, 486 individuals were retained for downstream analyses (Table 1). The quality filtering procedure allowed retaining a total of 52,174 SNPs and 11,321 SNPs after keeping only a single SNP per locus (see Supporting Information Table S1, for details). SNPs were distributed along all 10 chromosomes (Supporting information Figure S1). Preliminary analyses did not show clustering patterns based on IBM (Supporting information Figures S2 and S3).

### 3.2 Detecting SNPs putatively under selection and defining data sets

To identify putatively neutral and selected loci, we used the filtered SNP data set (11,321 SNPs). BAYESCAN identified 8,597, 20 and 2,704 SNPs being putatively neutral, under divergent selection and under balancing selection, respectively. ARLEQUIN identified 11,310 SNPs being putatively neutral, 11 SNPs putatively under divergent selection and no SNPs under balancing selection, while OutFLANK detected nine outliers (i.e., SNPs putatively under divergent selection). In total, 8,594 were identified to be putatively neutral using both BAYESCAN and ARLEQUIN. The evaluation of LD between markers identified 380 pairs of strongly linked (*R* ^2^ value ≥ 0.8) SNPs. By keeping only one of the strongly linked SNPs, we removed 348 SNPs, thus retaining 8,246 SNPs for the putatively neutral data set. Combining BAYESCAN, ARLEQUIN and OutFLANK, 23 SNPs were identified as being putatively under divergent selection. Of these SNPs, six were detected by all three methods, constituting the putatively adaptive data set.

### 3.3 Genetic diversity

The number of monomorphic and polymorphic loci calculated using the haplotype data set (comprising 4,307 loci) ranged from 181 (4%) to 364 (8%) and 4,126 (94%) to 3,943 (90%), respectively, between sampling sites, and 2% of reads were tagged as consensus (Table 2). The average *P*_i_ values ranged from 5.46 x 10^‐4^ (RIC) to 5.99 x 10^‐4^ (MAL) between sampling sites (Table 2), and 44.9% of the pairwise comparisons between sampling sites were significant (Supporting Information Table S2). Using the full 11,321 SNP data set, mean observed (*H*_o_) and mean expected heterozygosity (*H*_e_) were similar between sampling sites. All *H*_o_ values were 0.23, and *H*_e_ ranged from 0.28 to 0.29 for nonimputed data (Table 2). *F*_IS_ inbreeding coefficient values ranged from 0.188 (COC) to 0.211 (CRB) for nonimputed data (Table 2). Estimations performed with imputed (using the previously described procedure) and nonimputed data were similar (Table 2).

**Table 2:** Descriptive statistics for each oyster sampling sites, including proportion and number of monomorphic, polymorphic and consensus loci, observed heterozygosity (*H*_o_), expected heterozygosity (*H*_e_), inbreeding coefficient (*F*_IS_) and nucleotide diversity (*P*_i_)

Sites	Proportion and number of loci			*H*_o_	*H*_e_	*F*_IS_	*P*_i_
	Monomorphic	Polymorphic	Consensus				
CRQ	0.04	0.94	0.02	0.23	0.29	0.206 (0.200 – 0.211)	5.98 x 10^−4^
	181	4,126	75	**0.22**	**0.28**	**0.205 (0.200 – 0.210)**	
MIS	0.05	0.94	0.02	0.23	0.29	0.211 (0.206 – 0.216)	5.90 x 10^−4^
	199	4,108	75	**0.22**	**0.28**	**0.210 (0.205 – 0.216)**	
SSI	0.06	0.92	0.02	0.23	0.29	0.196 (0.190 – 0.201)	5.90 x 10^−4^
	283	4,024	75	**0.23**	**0.28**	**0.192 (0.187 – 0.198)**	
INK	0.05	0.93	0.02	0.23	0.29	0.198 (0.193 – 0.204)	5.78 x 10^−4^
	218	4,089	75	**0.23**	**0.28**	**0.197 (0.191 – 0.202)**	
TAB	0.06	0.92	0.02	0.23	0.29	0.202 (0.197 – 0.208)	5.58 x 10^−4^
	262	4,045	75	**0.22**	**0.28**	**0.201 (0.196 – 0.206)**	
MIR	0.08	0.90	0.02	0.23	0.28	0.196 (0.191 – 0.201)	5.49 x 10^−4^
	364	3,943	75	**0.23**	**0.28**	**0.194 (0.189 – 0.200)**	
RIC	0.08	0.90	0.02	0.23	0.28	0.202 (0.197 – 0.208)	5.46 x 10^−4^
	342	3,965	75	**0.22**	**0.28**	**0.200 (0.195 – 0.206)**	
BOU	0.05	0.93	0.02	0.23	0.29	0.203 (0.197 – 0.208)	5.77 x 10^−4^
	215	4,092	75	**0.22**	**0.28**	**0.201 (0.195 – 0.206)**	
COC	0.06	0.92	0.02	0.23	0.29	0.191 (0.185 – 0.196)	5.78 x 10^−4^
	259	4,048	75	**0.23**	**0.28**	**0.188 (0.182 – 0.193)**	
SHD	0.05	0.94	0.02	0.23	0.29	0.192 (0.187 – 0.197)	5.93 x 10^−4^
	202	4,105	75	**0.23**	**0.28**	**0.190 (0.185 – 0.195)**	
SHM	0.05	0.94	0.02	0.23	0.29	0.211 (0.206 – 0.217)	5.93 x 10^−4^
	206	4,101	75	**0.22**	**0.28**	**0.210 (0.204 – 0.215)**	
CRB	0.07	0.92	0.02	0.23	0.29	0.214 (0.208 – 0.219)	5.91 x 10^−4^
	293	4,014	75	**0.22**	**0.28**	**0.211 (0.205 – 0.217)**	
MAL	0.05	0.94	0.02	0.23	0.29	0.210 (0.204 – 0.215)	5.99 x 10^−4^
	208	4,099	75	**0.22**	**0.28**	**0.207 (0.201 – 0.212)**	

Note

Values in bold indicate *H*_o_, *H*_e_ and *F*_IS_ calculated on the imputed data set.

### 3.4 Genetic differentiation, population structure and population assignment

**Figure 2:**
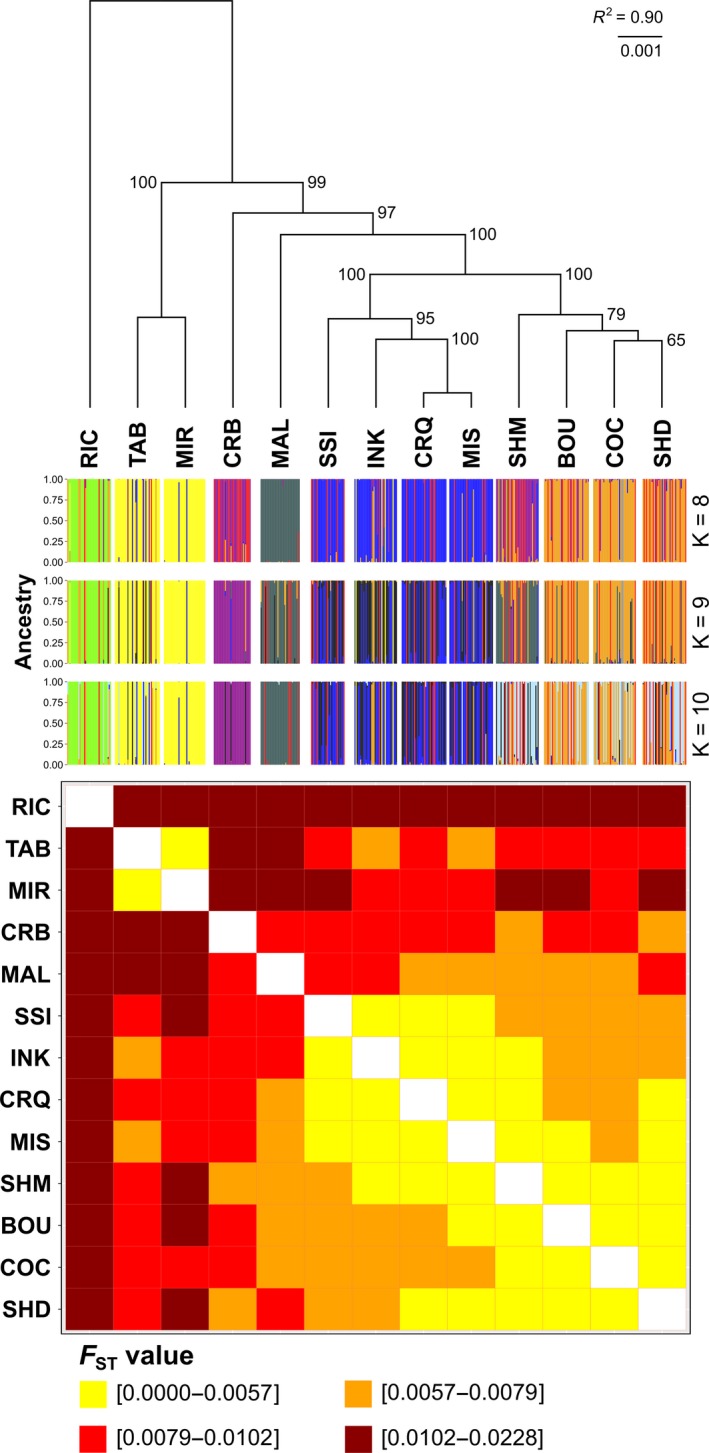
UPGMA dendrogram based on pairwise *F*_ST_ values among 13 eastern oyster sampling sites with bootstrap values based on 1,000 replicates, clustering of individuals identified by DAPC for *K* = 8 to 10 and heatmap based on pairwise *F*_ST_ values among the 13 sampling sites. All analyses are based on 8,246 neutral SNPs. Abbreviations of sampling sites are presented in Table 1

All pairwise comparisons of *F*_ST_ between sites were significantly different from zero (95% CI), except for CRQ and MIS. Missing data were thus imputed using CRQ‐MIS as a single group and all other sampling sites as unique groups. Pairwise estimates of *F*_ST_ using the putatively neutral data set (8,246 SNPs) between sampling sites ranged from 0.0007 (CRQ‐MIS) to 0.0228 (MAL‐RIC) (mean = 0.0090; Figure 2; Supporting Information Table S3). All 95% CIs excluded zero, and all corrected *p*‐values were <0.0001 except for CRQ‐MIS (*p* = 0.0246). The results of the Mantel test showed a weak but significant pattern of IBD (adjusted *R* ^2^ = 0.021, *p* = 0.039; Supporting Information Figure S4). The dendrogram revealed significant groupings of sampling sites (*R* ^2^ = 0.90, *p* = 0.001) that were highly supported with bootstrap support varying between 65% and 100% (median = 99.5%) (Figure 2).

Results from the DAPC analysis revealed a clustering pattern concordant with that based on genetic differentiation. Six genetic clusters were identified based on the lowest BIC value (Supporting Information Figure S5), though results associated with K values between 8 and 10 seemed more plausible according to the spatial distribution of sampling sites (Figure 2; Supporting Information Figure S6). Globally, results from genetic differentiation and clustering analyses suggested the occurrence of six clusters: (a) CRQ‐MIS‐SSI‐INK, (b) TAB‐MIR, (c) RIC, (d) BOU‐COC‐SHD‐SHM, (e) CRB and (f) MAL (Figure 2; Supporting Information Figure S6). The hierarchical AMOVA conducted using 12 populations (sampling sites with pairwise *F*_ST_ values <0.001 were considered as a single population) and the six clusters showed that 0.4% of the genetic variation could be explained by populations (*p *<0.0001) and 0.6% by clusters (*p* <0.0001) (see Table 3).

**Table 3:** Analysis of molecular variance (AMOVA) among oyster sampling locations

Source of variation	Percentage of variation	*F*_statistic_	*SD*	*p*‐value
Between groups	0.6	*F*_CT_ = 0.006 (0.006–0.007)	<0.001	<0.001
Between populations within group	0.4	*F*_ST_ = 0.004 (0.003–0.004)	<0.001	<0.001
Among individual within sampling locations	99.0	*F*_IS_ = 0.210 (0.213–0.223)	0.002	–

**Figure 3:**
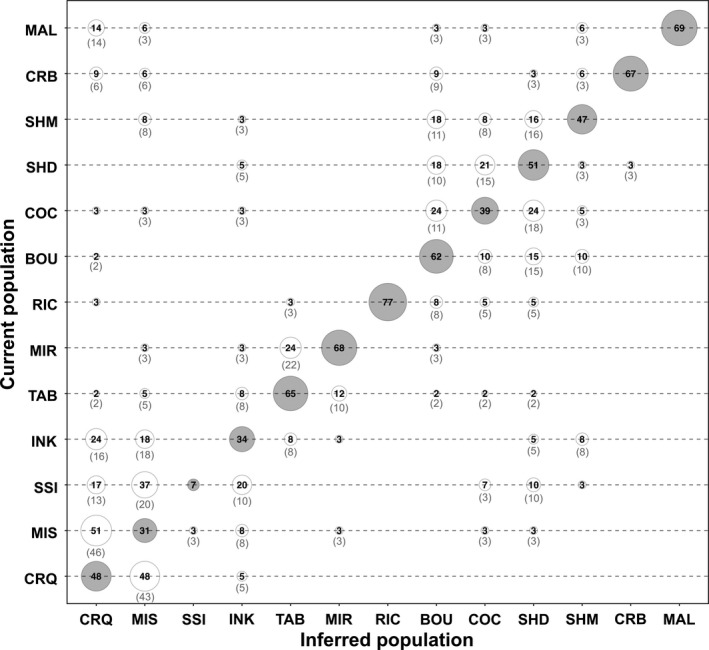
Assignment of individual oysters to their original (grey circles) or another sampling site (white circles). Numbers in circles represent the percentage of individuals from a sampling sites assigned to another. Circle diameter is proportional to percentage. Numbers in parenthesis represent the percentage of individuals from a “current population” tagged as migrants from a “inferred population”

Individual assignment success was highly variable between sampling sites. The proportion of correct assignment ranged from 7% (SSI) to 77% (RIC), and 52% of individuals was correctly assigned to their sampling sites (Figure 3). However, a higher proportion (77%) of individuals were correctly assigned when considering the six genetic clusters (Supporting Information Figure S7). Standardizing sampling size to *n* = 33 increased the assignment success to 81%, and a more uniform assignment success ranging from 73% (RIC) to 94% (CRB) (Supporting Information Figure S7). In all analyses, the majority of the incorrectly assigned individuals were tagged as migrants (Figure 3, Supporting Information Figure S7).

### 3.5 Association between spatial structure, environmental factors and genetic variation

**Figure 4:**
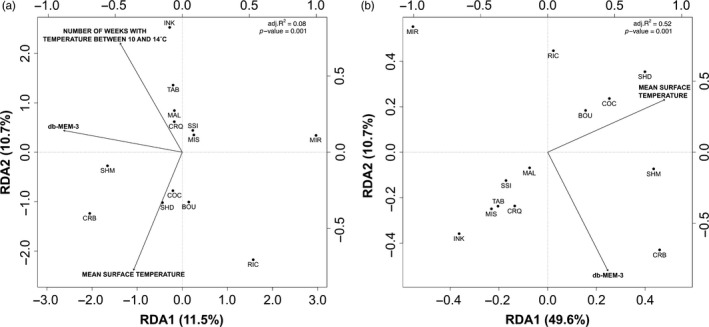
Redundancy analysis (RDA) for analyses performed on the 8,246 neutral SNPs (a) and the six loci putatively under divergent selection (b). Arrows represent significant spatial (dbMEMs) and environmental variables on axes 1 and 2. Sampling sites are represented by black filled circles. PC factors are positioned according to the top and right axes. Abbreviations of sampling sites are presented in Table 1

For the first RDA using putatively neutral genetic variation (8,246 SNPs), following the *ordistep* procedure, a single spatial vector (dbMEM‐3) and two temperature variables (number of weeks with temperature between 10 and 14°C and mean surface temperature) were selected for the RDA. The global RDA was highly significant and explained 7.7% of the genetic variation (*p* <0.001; Table 4; Figure 4). When we controlled for the effect of environmental variables in a partial RDA, the spatial dimension of the RDA was marginally significant and explained 3.1% of the genetic variation (*p* <0.083; Table 4). The environmental dimension was significant and explained 4.9% of the genetic variation after controlling for the spatial variable (*p* <0.015; Table 4).

**Table 4:** Redundancy analysis (RDA) results for potentially neutral and adaptive SNP data sets

Data set	Variable types	Significant variables	Adjusted *R* ^2^	*p*‐value
8,246 neutral SNPs	Global	–	0.077	<0.001
	Spatial	dbMEM−3	0.031	0.083
	Environmental	Mean surface temperature	0.049	0.015
		Number of weeks with temperature between 10 and 14°C		
Six SNPs potentially under selection	Global	–	0.524	<0.001
	Spatial	dbMEM−3	0.121	0.054
	Environmental	Mean surface temperature	0.386	<0.001

Note

Significance of the global model and significance of each variable in the partial RDA were evaluated using an ANOVA (10,000 permutations).

For the RDA based on the six SNPs identified as being putatively under divergent selection, following the *ordistep* procedure, a single geographic vector (dbMEM‐3; Table 4) and a single temperature variable (mean surface temperature) were selected for the RDA. The global RDA was highly significant and explained 52.4% of the genetic variation at these six outliers (*p* <0.001; Table 4; Figure 4). When we controlled for the effect of the environmental variables in a partial RDA, the spatial dimension of the RDA was marginally significant and explained 12.1% of the genetic variation (*p* = 0.054; Table 4), and the environmental dimension explained 38.6% of the genetic variation after controlling for the spatial distribution (*p* <0.001; Table 4).

**Figure 5:**
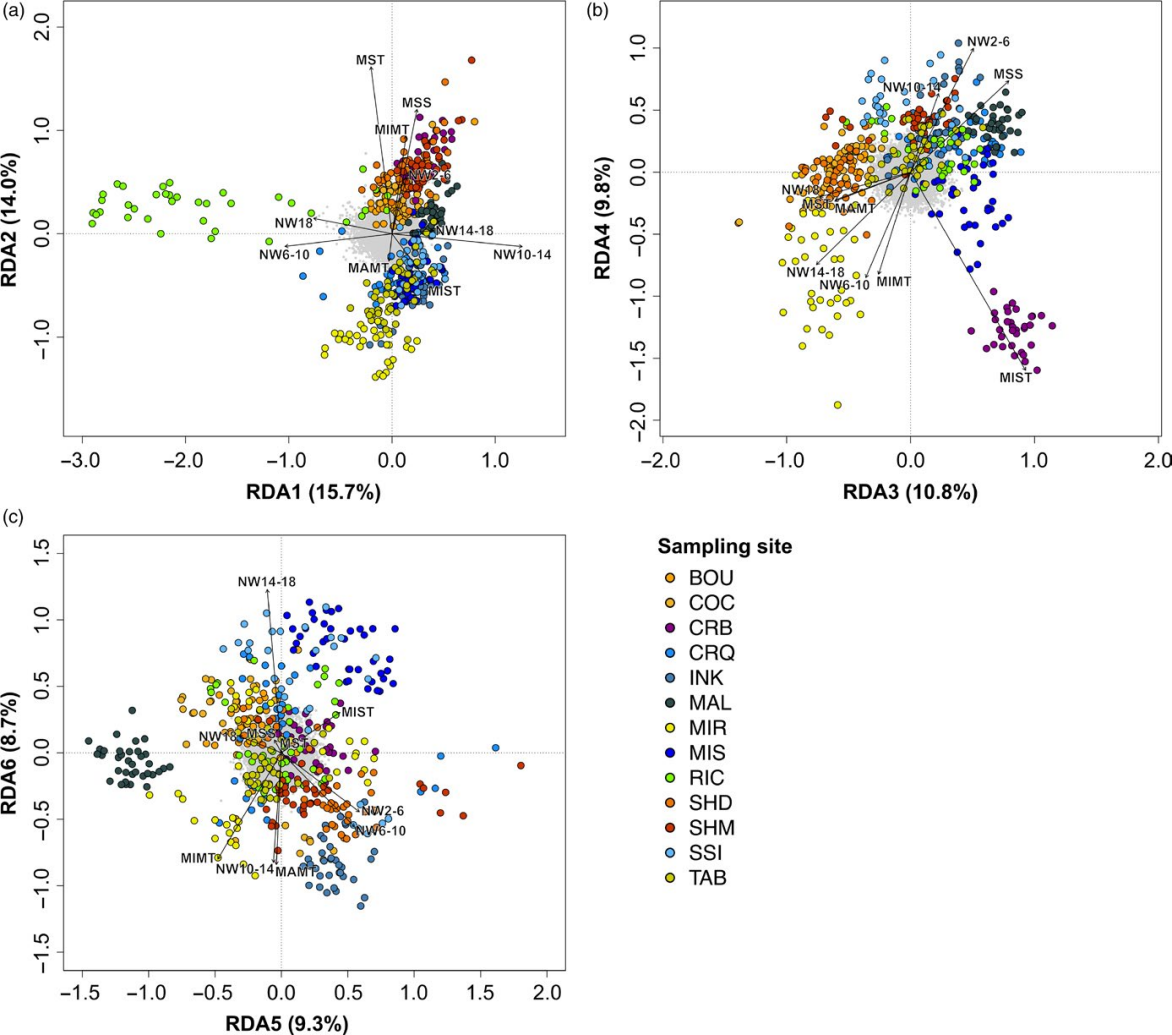
Redundancy analysis (RDA) for polygenic adaptation analyses performed using the 11,321 SNPs on significant axes, (a) axes 1 and 2, (b) axes 3 and 4, and (c) axes 5 and 6. Arrows represent environmental variables (MSS: mean surface salinity, NW2‐6: number of weeks with temperature between 2 and 6°C, NW6‐10: number of weeks with temperature between 6 and 10°C, NW10‐14: number of weeks with temperature between 10 and 14°C, NW14‐18: number of weeks with temperature between 14 and 18°C, NW18: number of weeks with temperature above 18˚C, MST: mean surface temperature, MIST: minimum surface temperature, MAMT: maximum monthly turbidity, MIMT: minimum monthly turbidity). Large coloured circles and small grey circles represent sampling sites and SNPs, respectively. Abbreviations of sampling sites are presented in Table 1

The global model of the multilocus genotype–environment RDA conducted using all 11,321 SNPs (one SNP per locus) to detect candidate loci under selection was significant (*p* <0.001). Results of the ANOVA used to test the significance of each constrained axis showed that the first six axes were significant (*p* <0.05). These axes explained a cumulative proportion of 68.3% of the genetic variation (Figure 5). The numbers of candidate SNPs associated with these significant axes were 93, 43, 42, 32, 38 and 39, respectively. A total of 47 candidate SNPs were associated with mean surface temperature, 38 with minimum surface temperature, 10 with number of weeks with temperature between 2 and 6°C, 30 with number of weeks with temperature between 6 and 10°C, 60 with number of weeks with temperature between 10 and 14°C, 27 with number of weeks with temperature between 14 and 18°C, 18 with number of weeks with temperature above 18°C, 8 with maximum monthly turbidity, 23 with minimum monthly turbidity, and 17 with mean surface salinity.

**Figure 6:**
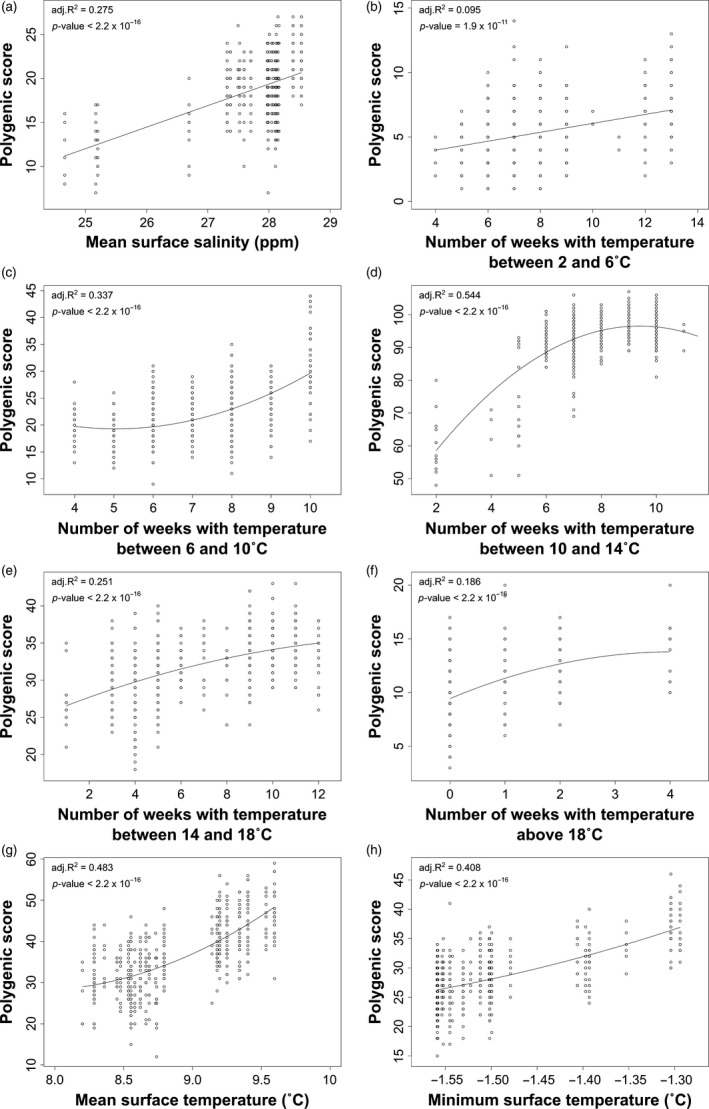
Correlations between additive individual polygenic score based on (a) mean surface salinity and 17 SNPs, (b) number of weeks with temperature between 2 and 6°C and 10 SNPs, (c) number of weeks with temperature between 6 and 10°C and 30 SNPs, (d) number of weeks with temperature between 10 and 14°C and 60 SNPs, (e) number of weeks with temperature between 14 and 18˚C and 27 SNPs, (f) number of weeks with temperature above 18°C and 18 SNPs, (g) mean surface temperature and 47 SNPs, (h) minimum surface temperature and 38 SNPs. Only significant correlations are presented. Correlation coefficient (*R* ^2^) and *p*‐values of the linear (a, b) or quadratic (c, d, e, f, g, h) models are presented for each variable

Correlations between polygenic scores and the corresponding environmental variable were all significant (*p* <0.001), with the exception of the relationship between polygenic scores and maximum (*p* = 0.344) and minimum (*p* = 0.707) monthly turbidity. The adjusted *R* ^2^ of the significant correlations ranged from 0.082 (minimum monthly turbidity) to 0.544 (number of weeks with temperature between 10 and 14°C) (Figure 6; nonsignificant correlations are presented in Supporting information Figure S8). The correlation between polygenic scores and both mean surface salinity and number of weeks with temperature between 2 and 6°C was best represented by a linear model, whereas a quadratic model better explained the relationship between polygenic scores and all other environmental variables.

### 3.6 Gene ontology

Of the markers identified as putatively under divergent selection, three SNPs were found using at least one genome scan method only, 258 using the RDA approach only and 20 using at least one genome scan method and RDA approach simultaneously. These 281 SNPs were distributed across the 10 chromosomes. Of all the sequences that were BLASTed on the protein sequences of the eastern oyster genome, 122 (40 loci, 122 SNPs) had significant hits and 36 SNPs, located on 21 loci and nine chromosomes, were nonsynonymous mutations (Supporting information Table S4). Results of GO on SWISS‐PROT showed that several genes containing nonsynonymous mutations were related to ion (metal and nonmetal) binding, ATP (adenosine triphosphate) binding, immunity and integral components of the cell. Of the proteins hit, six were categorized as “uncharacterized protein” (Supporting information Table S4).

## 4 DISCUSSION

Despite its economic and ecological importance, we know relatively little about levels of genetic structure and diversity in the eastern oyster. As a first step towards addressing this gap, we used RADSeq to document patterns of population structure between natural populations across its northern range limit. Contrary to our expectation that oyster populations in the study area would be genetically homogeneous due to larval dispersal, mass historical transfers and ongoing juvenile oyster translocations, our findings reveal a considerable degree of population genetic structure. Using genome scans and multivariate methods based on environmental associations, we identified putative targets of “monogenic” and “polygenic” selection, indicating that at least some of this variation is likely to be adaptive. In addition, our results also are consistent with an influence of spatial distribution and environmental factors, especially temperature, in shaping both neutral and adaptive genetic variation.

### 4.1 Genetic diversity of oysters in the studied region

Genetic diversity of the studied populations was not excessively low despite the historical decline of populations in our study area. Inbreeding levels (*F*_IS_) were positive, but were not as high as observed in other oyster species, such as the black‐lip pearl oyster using SNPs (*F*_IS_ > 0.5; Lal et al., 2017). Mean heterozygosity (*H*_o_ and *H*_e_) was slightly lower than or in the same range as what has been observed using SNP data sets in Pacific (*H*_o_ = 0.289–0.310, *H*_e_ = 0.276–0.315; Song, Li, Zhong, Kong, & Yu, 2017) and European oysters (*H*_o_ = 0.315–0.354, *H*_e_ = 0.305–0.320; Gutierrez et al., 2017) in their native ranges, and in the same range as in great scallops (*H*_o_ = 0.22; Vendrami et al., 2017). Results of nucleotide diversity (*P*i) suggest that RIC and MIR‐TAB have less diversity than MAL, CRQ‐MIS‐SSI, SHD‐SHM and CRB, which could potentially reflect a bottleneck in these clusters of populations. However, our results do not suggest that the studied natural populations suffer from a high level of inbreeding.

Heterozygote deficiencies are a well‐known feature of marine bivalves including the genus *Crassostrea* (Astanei, Gossling, Wilson, & Powell, 2005; Gaffney, 1990; Launey, Ledu, Boudry, Bonhomme, & Naciri‐Graven, 2002; Naciri et al., 1995; Raymond et al., 1997). Our observation of a heterozygote deficiency in all studied sites (i.e., positive *F*_IS_ values across sites, Table 2) corroborates this view. Oysters (and other bivalves) are known to have a high genetic load and exhibit segregation distortions (Bucklin, 2002; Launey & Hedgecock, 2001; Plough, 2016). This could be explained by a large load of deleterious recessive mutations, which is expected in highly fecund species for which great number of cell divisions is needed to produce gametes (Hedgecock et al., 2005; Williams, 1975), and an associated selection against these deleterious mutations (Bierne, Tsitrone, & David, 2000; Launey & Hedgecock, 2001; McGoldrick & Hedgecock, 1997; McGoldrick, Hedgecock, English, Baoprasertkul, & Ward, 2000; Yu & Guo, 2003). Selection against deleterious alleles throughout the genome could negatively affect heterozygosity in particular regions through linked loci (Charlesworth, Morgan, & Charlesworth, 1993; Wang & Hill, 1999; Zouros, Singh, & Miles, 1980). Mallet, Zouros, Gartner‐Kepkay, Freeman, and Dickie (1985) suggested that selection (against heterozygotes) acting in the larval stage could be responsible for heterozygote deficiency in marine bivalves. In addition, sweepstakes reproductive success could contribute to heterozygote deficiency by inducing population substructure among different age classes and creating a temporal Wahlund effect***. ***On the other hand, heterozygote fitness advantage (overdominance) has been suggested in eastern oysters (Hu, Lutz, & Vrijenhoek, 1993; Singh, 1982; Singh & Zouros, 1981; Zouros & Foltz, 1983; Zouros, Singh, Foltz, & Mallet, 1983), which may act as a stabilizing force in maintaining heterozygosity.

### 4.2 Evidence for significant population structure

Despite the expected genetic homogeneity of oysters due to historical translocations in the studied region and the high dispersal potential of oyster larvae (Vercaemer et al., 2010), we found a clear pattern of population structure reflected by both significant geographic clustering of sampling sites and significant isolation by distance. Indeed, the extent of genetic differentiation is relatively pronounced in comparison with other marine invertebrates from the same region. Within a similar geographic region, previous studies revealed average *F*_ST _values (neutral loci) of 0.0018 and 0.003 in American lobster (Benestan et al., 2015) and in Sea scallop (Van Wyngaarden et al., 2017), respectively. These values are considerably lower than the average *F*_ST_ value of 0.009 that we observed in our populations but comparable to that reported for the acorn barnacle (*Balanus balanoides*) in the same region (0.010) (Dufresne, Bourget, & Bernatchez, 2002).

Several features of oyster populations may lend themselves to the observed degree of population differentiation. Most importantly, oysters are not directly open sea organisms, but rather inhabit semi‐closed estuaries and form discontinuous populations. Globally, six clusters were distinguishable, and each cluster is formed from geographic “neighbours.” The four northern sampling sites in NB (CRQ, MIS, SSI and INK) formed a single cluster, followed by two central NB populations (TAB and MIR). Richibucto (RIC) formed a distinct cluster, and the four southern NB sampling sites (BOU, COC, SHD and SHM) formed another cluster. The last two sampling sites, outside of NB (CRB and MAL), each formed independent clusters. Significant population structuring in the system was also supported by the relatively strong assignment success of 77% among the six resolved genetic clusters. Using balanced sampling sizes led to a higher and more uniform assignment success between clusters, corroborating the importance of sample size in assignment analyses.

While each regional cluster was formed of geographic neighbours, the overall pattern of genetic structure does not have a simple relationship to physical distance. For example, the northern NB cluster and the southern NB cluster form a group with Malpeque (Figure 2), to the exclusion of the other NB populations in the centre of the province. Notably, Richibucto (RIC) appears to be highly divergent from every other population, with pairwise *F*_ST_ values ranging from 0.0157 (RIC‐SHD) to 0.0228 (RIC‐MAL), well above the global average pairwise *F*_ST_ of 0.009.

### 4.3 Environmental effects on genetic variation

Using monogenic and polygenic approaches, we identified environmental factors that are correlated with changes in allele frequencies for both putatively neutral and adaptive loci in our study area. In particular, our results suggest that temperature has a significant impact on both neutral and putatively adaptive genetic variation throughout the system, with two temperature variables (i.e., number of weeks between 10 and 14°C and mean surface temperature) being significantly correlated with neutral genetic variation using the RDA approach. For adaptive genetic variation, both temperature and salinity appear to be important based on the results of the monogenic and polygenic approaches.

Environmental variables can shape neutral variation through their impact on gene flow, for example, by acting as barriers to long‐distance dispersal. Oysters disperse uniquely during the larval period, a particularly vulnerable life‐history stage that is characterized by extremely high mortality rates (Dekshenieks, 1996; He et al., 2012). Larval traits such as development time, propensity to settle and swimming speed are directly related to temperature (Devakie & Ali, 2000; Hidu & Haskin, 1978; Johnson, 1957; Shumway, 1996). At their northern range limit, oysters become quiescent for almost half the year, from mid‐November to early May (Comeau, Mallet, Carver, Nadalini, & Tremblay, 2017), so newly settled spat have a short window of time to accumulate energy reserves before the winter. As late recruitment has been suggested to cause high winter mortality (Mallet & Haley, 1983), migrants arriving later from neighbouring bays may be at a disadvantage.

Several putatively adaptive SNPs were highly correlated with temperature variables, both as contributors to monogenic and polygenic selection. Oyster metabolic rate and life cycle events, like gonad development and spawning, are temperature‐dependent (Barber, Ford, & Wargo, 1991; Gabbott, 1975; Mann, Morales‐Alamo, & Rainer, 1994; Price & Maurer, 1971; Thompson, Newell, Kennedy, & Mann, 1996). Temperature has also been linked to life‐history variation in other parts of its range (Buroker, 1983; Dittman, Ford, & Haskin, 1998; King, Ward, & Zimmerman, 1994). Moreover, temperature has previously been suggested to be of adaptive importance in other oyster species (Buroker, 1983; Eastern oyster Biological Review Team, 2007; King et al., 1994) and in other marine invertebrates (Benestan, Quinn et al., 2016; Palumbi, Barshis, Traylor‐Knowles, & Bay, 2014; Pespeni & Palumbi, 2013). Given the link between temperature and life‐history characteristics such as feeding, growth, maturation and spawning timing in oysters (Davis & Calabrese, 1964; Eastern oyster Biological Review Team, 2007; Kennedy, Newell, & Ebel, 1996; Shumway, 1996; Spires, 2015) and other marine invertebrates (Davis & Calabrese, 1964; Farhadian, Yusoff, & Arshad, 2014; Sastry, 1979; Stommes, Fieber, Beno, Gerdes, & Capo, 2005), and that our study area represents the northern range limit for eastern oysters, it seems particularly likely that temperature is a selective agent acting on eastern oysters, as reported previously for the acorn barnacle from the same region (Véliz, Bourget, & Bernatchez, 2004).

Mean surface salinity also had a significant correlation with a set of SNPs, suggesting polygenic selection related to salinity. Along with temperature, salinity has been shown to be a major driver of ecological processes in the eastern oyster, affecting growth, survival and recruitment (Davis & Calabrese, 1964; La Peyre, Eberline, Soniat, & Peyre, 2013; Petes, Brown, & Knight, 2012; Pollack, Kim, Morgan, & Montagna, 2011; Soniat, Klinck, Powell, & Hofmann, 2012). Adaptation related to salinity tolerance has already been suggested in other studies (Buroker, 1983; Eierman & Hare, 2013; King et al., 1994; Newkirk, 1978).

Finally, temperature and salinity influence disease dynamics in oysters. As filter feeders, oysters are continuously exposed to pathogens (Gestal et al., 2008; Zhang et al., 2012) and some diseases, such as Dermo and MSX, are known to be more prevalent and infectious at higher temperature and salinity (Breitburg et al., 2015; Cook, Folli, Klinck, Ford, & Miller, 1998; Ford, 1992; La Peyre, Casas, Gayle, & Peyre, 2010; Petes et al., 2012). While these diseases are not present in our study area, it is important to note that the causative agent of Malpeque disease has never been identified (DFO, 2017) and that it is still present in the study area, as experimentally demonstrated by massive mortalities following transfer of naïve oysters to the Gulf of Saint Lawrence (McGladdery & Zurbrigg, 2006). Malpeque disease appears to be restricted to higher salinity regions of Atlantic Canada (DFO, 2017; Eastern oyster Biological Review Team, 2007). Thus, while oyster populations in the Gulf of Saint Lawrence do not suffer currently large mortalities, Malpeque disease may still exert an influence on extant oyster population structure and this may be mediated by abiotic variables, as for other diseases (Bergquist, Hale, Baker, & Baker, 2006; Burreson & Ragone‐Calvo, 1996; Eastern oyster Biological Review Team, 2007; Hofmann et al., 2009; Mann, Southworth, Harding, & Wesson, 2009; McGladdery & Zurbrigg, 2006; McGladerry & Bower, 1999).

### 4.4 Contemporary demographic history and anthropogenic influences on population structure

In addition to the spatial and environmental factors uncovered in our analyses, the particular demographic history (i.e*.*, recent mass mortality due to Malpeque disease) and contemporary anthropogenic influences (e.g., movement of adults and spat by industry) of oysters in our study area may have impacted extant population genetic structure. Indeed, Malpeque disease is the primary reason oyster populations in the Gulf of Saint Lawrence were assumed to be genetically homogeneous. In New Brunswick, Malpeque disease occurred in the 1950s, with massive transplantation of PEI oysters during the 50s and 60s. Oysters in our study area can spawn in the year following settlement, although the number of gametes is very small initially and increases with oyster size/age (Choi, Lewis, Powell, & Ray, 1993). With a conservative 2‐year generation time, oyster populations have had no more than 25 generations since the end of rehabilitation transfers, a period of time during which oyster translocations have been continuing. Yet, early experimental evidence has suggested that some oyster populations in the Maritimes have been differentiated for life‐history traits for several decades. For example, Mallet and Haley (1984) performed inter‐population crosses using oysters from throughout NB and found evidence for additive and dominance effects of population on larval and juvenile traits. That oyster populations in our study area show significant population structure suggests either rapid population diversification since the rehabilitation transfers or the persistence of ancestral population structure.

Certain features of oyster biology may lead to relatively rapid diversification, especially sweepstakes reproductive success (SRS), which has often been suggested as a major feature of highly fecund marine species (including oysters) subject to high larval mortality (reviewed in Hedgecock and Pudovkin (2011)). It implies a large effect of genetic drift and lower genetic diversity than expected given the large census populations size (Hedgecock & Pudovkin, 2011), and has been suggested as a characteristic of certain oyster species (Boudry, Collet, Cornette, Hervouet, & Bonhomme, 2002; Hedgecock, 1994; Lallias, Taris, Boudry, Bonhomme, & Lapegue, 2010; Sun & Hedgecock, 2017). Some studies failed to reveal strong events of SRS in eastern oyster (He et al., 2012; Rose, 2008; Rose, Paynter, & Hare, 2006); however, SRS is difficult to test as it may fluctuate spatially and temporally, making it difficult to reveal locally or to reject at the species level (Hedgecock et al., 2007; Hedgecock & Pudovkin, 2011; Taris, Boudry, Bonhomme, Camara, & Lapègue, 2009). Nevertheless, SRS seems unlikely to account for the magnitude of population differentiation we observed, especially as there is no obvious difference between the source population (MAL) and other populations with respect to genetic diversity parameters, which would be expected if pervasive genome‐wide population bottlenecks were a major contributor to diversification. While variance in the reproductive success of broadcast spawning oysters is undoubtedly high, the large‐scale nature of the transfers (several tons per bay) may have prevented strong founder effects, even if the effective population size (Ne) may be much smaller than census size (Nc) (Hauser & Carvalho, 2008). As a reproductive‐mediated form of random drift, SRS is also not a plausible candidate for the apparent pattern of adaptive differentiation in our study area.

Rather, it is plausible that extant oyster populations reflect a mixture of “ancestral” populations and introgressed alleles from PEI oysters. In northern and southern NB, oyster populations seem to be more related to MAL (in PEI) and thus probably more related to the resistant oysters used for repopulation. However, the populations from central NB (i.e., TAB, MIR and RIC) and NS (CRB) do not cluster with the “Malpeque clade.” A possible explanation for this result is that these oysters may have independently acquired resistance to Malpeque disease or that there was limited introgression of resistance‐related genes of PEI oysters in these populations, due either to the presence of a larger number of oysters having survived the Malpeque outbreak (e.g., in low‐salinity refugia) or to countervailing local selection pressure. As our results suggest that environmental conditions are at least partially responsible for the observed neutral and adaptive genetic variation, it is possible that a part of the “ancestral” genetic variation was preserved through selection.

Evaluating the impact of ongoing human‐induced movement is also very difficult with our data set. Within NB, transfer permits are issued with an emphasis on mitigating the risks associated with aquatic invasive species and diseases, but there is no tracking of oyster movements by industry. The oyster aquaculture industry is spread out throughout NB, with aquaculture leases in all of the defined regional clusters. Oyster movements frequently occur between the northern and southern NB clusters, and between the Richibucto Estuary and southern NB, though most oysters tend to be moved within a cluster due to the logistics of wild spat collection (Sylvio Doiron, personal communication). Importantly, the scope of activities carried out by the oyster aquaculture industry in NB has been relatively limited until the last 10 years. We were careful to avoid aquaculture leases in our sampling and sampled mature oysters, most of which are likely to be more than 5 years old, so it is also distinctly possible that insufficient time has passed for widespread hybridization to be detectable.

### 4.5 Highlights from gene ontology

Of the loci identified to be putatively under divergent selection, 21 (mostly associated with temperature variables) were found to be located within genes with at least one nonsynonymous mutation. Among the gene functions identified, several were related to ATP and ion binding, and one was related to immunity. Mitochondrial oxidative phosphorylation, in which energy is stored as ATP, is the major aerobic energy production process (Mazat, Ransac, Heiske, Devin, & Rigoulet, 2013). Oysters are well adapted to frequent hypoxia–reoxygenation events and some adaptations involve deviations of ATP production and consumption mechanisms (Abe, Yoshikawa, Sarower, & Okada, 2005; Ivanina, Kurochkin, Leamy, & Sokolova, 2012). Temperature can considerably affect oxygen consumption, energy metabolism, mitochondrial efficiency and response to hypoxia–reoxygenation, and thus ATP‐related functions (Abele, Heise, Pörtner, & Puntarulo, 2002; Chamberlin, 2004; Cherkasov, Biswas, Ridings, Ringwood, & Sokolova, 2006; Ivanina et al., 2012; Sokolova, 2004). As several loci (containing nonsynonymous mutations) located within genes known to have ATP‐related functions were also associated with temperature, it is possible that adaptive divergence observed in the studied system could be linked with thermal adaptation and energetic metabolism, as revealed in other *Crassostrea* species (Li, Li, Song, Wang, & Zhang, 2017) and other molluscs (Sokolova & Pörtner, 2001; Tomanek & Zuzow, 2010). Moreover, ATP is a critical component (see Coyne, 2011) of the energetically expensive immune system of molluscs (Gestal et al., 2008; Sokolova, 2009).

In oysters, energy‐demanding processes like gametogenesis and spawning, which are triggered (not exclusively) by high temperatures (Ruiz, Abad, Garcia‐Martin, & Sanchez Lopez, 1992; Steele & Mulcahy 1999; Dridi, Romdhane, & Elcafsi, 2014; Ubertini et al., 2017), can be coupled with disease episodes, which generally occur in the summer months. As such, if the immunity response is depressed while energy reserves are low, disease outbreaks can have devastating effects (Coyne, 2011; Delaporte et al., 2006; Huvet et al., 2004; Li, Qin, Li, & Benkendorff, 2010; Pouvreau et al., 2003). The strictly innate nature of immunity response in oysters makes it particularly prone to selection, which has been suggested in other studies (Dégremont et al., 2003; Huvet et al., 2004; Samain et al., 2007).

In summary, despite the possible influence of Malpeque disease and population translocations, it appears that eastern oysters are still characterized by relatively pronounced population structure (in relative terms compared to other marine species), which could be associated with differential selection across temperature and salinity regimes. However, further investigation is clearly needed to elucidate the physiological basis for putative adaptive divergence in the eastern oyster.

### 4.6 Implications for eastern oyster management and future research directions

Considering the ecological and economical importance of the eastern oyster in Canada, studying the extent of genetic divergence between populations and the factors responsible for the observed differentiation is a crucial step towards better management. Despite massive mortalities and human‐mediated translocations, our results demonstrate a clear pattern of genetic divergence related, at least partially, to spatial proximity in oyster populations within the studied region. Moreover, we found evidence that environmental conditions (primarily temperature, but also salinity) were associated with putatively adaptive genetic variation in the system. The presence of local adaptation may argue for management measures aimed at limiting introgression from divergent populations (Conover, 1998; do Prado et al., 2018), including limiting the scope of transfer activities carried out by industry, and therefore, a major outstanding question for oyster management is determining to what extent wild populations are locally adapted.

While we found correlative evidence that environmental variables were linked to changes in allele frequencies at both putatively adaptive and neutral loci, much work remains on elucidating the causative links and relevant scales for selection. For example, we relied on a government database (Dutil et al., 2012) for the environmental variables that provided environmental data in the coastal waters surrounding our study populations. We did not have direct access to environmental data within the estuaries themselves. Estuaries are known to be extremely heterogeneous environments, and oysters are found in a broad range of habitats, spanning large gradients in temperature and salinity (see Eastern oyster Biological Review Team, 2007). Finer resolution environmental data and within‐bay sampling may reveal further patterns of selection and differentiation, which may impact the broad‐scale correlation observed here. For example, Newkirk (1978) found evidence of differentiation with the Richibucto (RIC) Estuary between high‐ and low‐salinity sites. Importantly, these differences persisted into the F2 indicating that parental environmental effects were not the sole driver of performance differences. Other environmental variables, such as pH, are highly important to calcifying bivalves (Gazeau et al., 2007; Liu et al., 2017; Miller, Reynolds, Sobrino, & Riedel, 2009; Zhao et al., 2017) but were not available to use in our analyses. Finally, while empirical studies have shown population effects on many aspects of eastern oyster performance and GxE interactions (Dittman et al., 1998; Frank‐Lawale, Standish, Allen, & Dégremont, 2014; Mallet & Haley, 1983; Newkirk, 1978), the demonstration of local adaptation, as opposed to balanced polymorphism maintained by spatially varying selection, is not straightforward (Sanford & Kelly, 2011).

This study aimed to provide a first step to understanding patterns of genetic diversity and population structure in eastern oysters from the Maritime provinces of Canada, and future research can elaborate on this work. Genetic patterns of differentiation in relation to environmental variables should be investigated at smaller spatial scales and with an increased sample size to determine whether genetic substructure exists. While this work may not be feasible across all bays, the presence of regional genetic clusters may suggest future empirical work should at least aim to include representatives from multiple genetic clusters. Temporal replication and single‐cohort sampling would be powerful tools in deciphering the contribution of SRS to shifts in allelic frequencies and diversification, as well as the presence of temporal fluctuations in selection. Admittedly also, RADSeq methods as used here only allow the screening of a subsample of the genome‐wide genetic variation within a given species. Therefore, future population genomic studies on this species would benefit from using a whole genome resequencing approach (Fuentes‐Pardo & Ruzzante, 2017).

It is important to elucidate the precise selective mechanism causing the observed pattern of differentiation before considering a change in the current management of oyster movements. Large‐scale historical transfers were successful in re‐establishing oyster populations, suggesting that current stocks are at least tolerably well adapted to their present environmental conditions. In addition, it is important to recall that the extant pattern of population differentiation is present despite ongoing movements by industry, though it may be too early to detect a genomic signature from the recent expansion of aquaculture activities in our data set. Ultimately, our data provide an intriguing snapshot of population genomic diversity in eastern oysters, which has uncovered patterns of genetic differentiation that warrant further investigation.

Understanding spatial patterns of adaptive genetic variation and associations with environmental variables has important implications for predicting how eastern oyster populations might respond to environmental change in the future. With climate change, the selective seascape for eastern oysters is likely to dramatically shift in the coming decades. Warmer and more acidified waters are expected to have a host of complex impacts, both direct (e.g., temperature stress) and complex (e.g., changing host–disease interactions, multiple environmental stressors). A better understanding of genotype–environment associations and the targets of selection in Maritime populations of the eastern oyster will contribute to mitigating these impacts and ensuring the long‐term viability of fishery and aquaculture activities.

## CONFLICT OF INTEREST

None Declared.

## Data Availability

Genomic data (all filtered markers, putatively neutral markers and markers putatively under divergent selection) and environmental data for the RDA are available on Dryad (https://doi.org/10.5061/dryad.g487s21). Raw demultiplexed DNA sequences are available on NCBI SRA (Project Accession Number: SRP169933).
