# Supplementary material for: Seascape genomics of eastern oyster (Crassostrea virginica) along the Atlantic coast of Canada
Source: Evol Appl. 2018 Dec 26;12(3):587–609. doi: 10.1111/eva.12741 (PMC6383708; doi:10.1111/eva.12741)

**Supplementary file 1** Detailed protocol of ddRAD-Seq library preparation protocol described by Peterson *et al*. (2012) modified to better suit oysters.

*Restriction enzyme digestion*

DNA samples were normalized to a concentration of 25 ng/µL and transferred to 96-well plates. Enzymatic digestion was performed in 50 µl volumes by digesting 1 µg of normalized genomic DNA from each sample in 1X cutsmart® buffer with 20 units of NsiI-HF (New England Biolabs, R3127S) and 20 units of MspI (New England Biolabs ,R0106S) restriction enzymes. Incubation was carried out at 37^o^C for 3 hours. The resulting DNA was purified using Agencourt AMPureXP beads (Beckman Coulter) following the manufacturer’s protocol and eluted in 60 µL of DEPC treated water. DNA samples were then randomized and transferred to new 96 well plates.

*P1 and P2-MspI adapter barcoding and multiplexing of samples*

All barcoded top and bottom strand adapters were ordered from IDT Technologies and corresponding top and bottom strands were annealed together as described by Etter *et al* (2011). In total, 62 different barcoded P1 adapters of 4, 5 or 6 nucleotides in length were used for this step. The P2-MspI adapters contained one of six unique 6 bp Illumina index sequences in the adapter and had an MspI DNA overhang to anneal to the digested DNA. Purified restriction digest products were ligated to their corresponding pre-annealed P1 and P2 adapters by combining the restricted DNA with 4.35 pmole of P1 adapter, 6.1 pmole of P2-MspI adapter, 1X Ligation Buffer, 1,000 units of T4 DNA Ligase (New England Biolabs, M0202S) and DEPC treated water to a final volume of 80 µL. Plates were incubated at room temperature overnight and inactivation was carried out by incubating the samples at 65^o^C for 10 minutes before performing another AMPureXP bead purification and eluted in 60 µL of sterile water. For each DNA plate, pooled samples were prepared by combining 50 ng of DNA, quantified with the Quant-it PicoGreen® dsDNA assay kit (Invitrogen) on an Mx300P (Agilent Technologies) qPCR machine, from each well containing the same P2-MspI index in a labelled microtube. The volume of these pooled samples was reduced using a SpeedVac concentrator (Eppendorf) to reach a final volume of 50 µL and re-purified with AMPureXP beads (Beckman Coulter) and eluted in 60 µL of sterile water.

*Size selection of DNA*

Automated size selection was performed using 30 µL of pooled DNA per lane of a Pippin Prep instrument (Sage Science) with 2% agarose dye-free gel cassettes following the manufacturer’s instructions for the elution of DNA fragments between 300 and 400 bp in length.

*RAD tag amplification and verification of DNA quality*

In order to assess the quality of each pool of samples, 2 µL of adapter ligated DNA was added to a mixture composed of 12.5 µL of 2x Phusion Mastermix (New England Biolabs M00532S), 10 pmole of primers PCR1, 10 pmole of the appropriate PCR2 primer and sterile water added to a final volume of 25 µL. Samples were incubated in a Mastercycler EP (Eppendorf) and PCR parameters consisted of an initial denaturation step at 98^o^C for 30s, followed by 18 cycles at 98^o^C for 10s, 55^o^C for 30s and 72^o^C for 30s. A final elongation step was performed at 72^o^C for 5 min. The amplified DNA was then loaded on a 1% agarose gel and electrophoresis was carried out at 100 volts. Staining of the DNA bands was done by adding GelRed (Biotium) to the agarose preparation and bands were visualised under UV light. After having confirmed that there was sufficient amplification in each sample and that the DNA fragments amplified were within the desired size range new PCR reactions were carried out. These new PCR reactions consisted of quadruplicate PCR reactions for each pool using 10 µL of DNA as template instead of 2 µL. Thermocycling parameters were the same as described above, however, only 15 cycles of PCR were performed instead of 18 in an effort to minimize artefacts and PCR duplicates. Next, replicate PCR reactions were pooled together and the resulting products were purified again using AMPureXP beads (Beckman Coulter) with a 1x volume of beads was in order to remove excess primers. The DNA was eluted in a final volume of 60 µL of sterile water. Samples were quantified with a Qubit® fluorometer (ThermoFisher) and normalized before pooling all individually indexed samples together for each of 5 HiSeq lanes (each HiSeq lane contained all 6 P2-MspI indices). To verify the size distribution of the DNA before sequencing, 1 µL of each sample was loaded onto an Experion 1K DNA Analysis Chips (Bio-Rad), following the manufacturer’s protocol. Samples were then sent out for sequencing on a HiSeq 2000 platform (Illumina). An internal PhiX control was added at a 15% concentration and clustering density was approximately 650k/mm^2^.

**References**

Etter, P. D., Preston, J. L., Bassham, S., Cresko, W. A., & Johnson, E. A. (2011) Local *de novo* assembly of RAD paired-end contigs using short sequencing reads. *PLoS One*, 6, e18561. https://doi.org/10.1371/journal.pone.0018561

Peterson, B. K., Weber, J. N., Kay, E. H., Fisher, H. S., & Hoekstra, H. E. (2012). Double Digest RADseq: An inexpensive method for *de novo* SNP discovery and genotyping in model and non-model species. *PLoS ONE*, 7, e37135. https://doi.org/10.1371/journal.pone.0037135

**Table S1** Number of markers (SNPs and loci) remaining after each major steps of filtering.

| **Filters** | **Number of remaining markers** | |
| --- | --- | --- |
|  | **SNPs** | **Loci** |
| **Stacks catalog** | - | 921 308 |
|  |  |  |
| **Populations output (r = 0.5, p = 7, m = 4)** | 46 3360 | 47 950 |
|  |  |  |
| **Genotype filters** |  |  |
| read.depth.threshold = 5 | - | - |
|  |  |  |
| **SNP and loci filters** | 52 174 | 11 347 |
| ind.threshold = 80% (one population can be lower) |  |  |
| population.maf.threshold = 0.05 |  |  |
| global.maf.threshold = 0.01 |  |  |
| het.threshold = 0.6 (one population can be higher) |  |  |
|  |  |  |
| **Keep 1 SNP per loci (max MAF)** | 11 347 | - |
|  |  |  |
| **Remove SNPs within motchondrial DNA** | 11 344 | - |
|  |  |  |
| **Keep only 1 SNP for SNPs with same position (sequencing and pipeline artefact)** | **11 321** | - |
|  |  |  |
| **Remove artefacts and add consensus loci** | **-** | **4 307** |
| Remove loci > 2 haplotypes in more than 5 individuals |  |  |
| Erase individual genotypes for loci > 2 haplotypes in a maximum of 5 individuals |  |  |
| Remove loci > 80% of missing data |  |  |
| Add concensus loci < 80% of missing data |  |  |
|  |  |  |

**Table S2** Pairwise comparison of nucleotide diversity (*P*i) among sampling sites using *t* test. *t* values and associated *P*-values are presented above and below the diagonal, respectively. Significant (*P* < 0.05) comparisons are in bold.

| **Sites** | CRQ | MIS | SSI | INK | TAB | MIR | RIC | BOU | COC | SHD | SHM | CRB | MAL |
| --- | --- | --- | --- | --- | --- | --- | --- | --- | --- | --- | --- | --- | --- |
| CRQ | -- | 0.846 | 0.851 | **2.249** | **4.484** | **5.420** | **4.936** | **-2.141** | -1.928 | 0.406 | 0.425 | -0.623 | -0.137 |
| MIS | 0.400 | -- | 0.034 | -1.555 | **4.096** | **-5.156** | **4.579** | -1.493 | -1.269 | -0.330 | -0.355 | 0.118 | 0.982 |
| SSI | 0.398 | 0.973 | -- | -1.452 | **3.892** | **-4.909** | **-4.417** | -1.413 | -1.198 | 0.350 | 0.374 | 0.144 | 0.983 |
| INK | **0.028** | 0.124 | 0.152 | -- | **2.728** | **3.854** | **3.451** | -0.147 | 0.056 | -1.575 | -1.688 | 1.402 | **-2.366** |
| TAB | **< 0.001** | **< 0.001** | **< 0.001** | **0.008** | -- | -1.107 | -1.217 | **2.206** | **2.350** | **3.609** | **3.857** | **3.514** | **4.562** |
| MIR | **< 0.001** | **< 0.001** | **< 0.001** | **< 0.001** | 0.272 | -- | 0.310 | **3.177** | **3.298** | **-4.457** | **-4.763** | **4.394** | **5.482** |
| RIC | **< 0.001** | **< 0.001** | **< 0.001** | **< 0.001** | 0.228 | 0.757 | -- | **2.990** | **3.106** | **-4.181** | **-4.399** | **4.101** | **5.006** |
| BOU | **0.035** | 0.140 | 0.162 | 0.883 | **0.031** | **0.002** | **0.004** | -- | -0.178 | -1.553 | -1.645 | -1.392 | **-2.251** |
| COC | 0.058 | 0.209 | 0.235 | 0.956 | **0.022** | **0.002** | **0.003** | 0.859 | -- | -1.369 | -1.448 | -1.204 | **-2.039** |
| SHD | 0.686 | 0.743 | 0.728 | 0.120 | **< 0.001** | **< 0.001** | **< 0.001** | 0.125 | 0.175 | -- | -0.003 | -0.191 | 0.528 |
| SHM | 0.672 | 0.724 | 0.709 | 0.096 | **< 0.001** | **< 0.001** | **< 0.001** | 0.104 | 0.152 | 0.998 | -- | -0.204 | 0.552 |
| CRB | 0.535 | 0.907 | 0.886 | 0.166 | **< 0.001** | **< 0.001** | **< 0.001** | 0.167 | 0.233 | 0.849 | 0.839 | -- | -0.746 |
| MAL | 0.892 | 0.330 | 0.329 | **0.021** | **< 0.001** | **< 0.001** | **< 0.001** | **0.027** | **0.045** | 0.599 | 0.583 | 0.458 | -- |
|  |  |  |  |  |  |  |  |  |  |  |  |  |  |

**Table S3** Pairwise estimates of fixation index (*F*_ST_) for 8 246 neutral SNPs (imputed data) between sampling sites. *F*_ST_ values and 95% confidence intervals (10 000 bootstraps) are presented above and below the diagonal, respectively.

| **Sites** | CRQ | MIS | SSI | INK | TAB | MIR | RIC | BOU | COC | SHD | SHM | CRB | MAL |
| --- | --- | --- | --- | --- | --- | --- | --- | --- | --- | --- | --- | --- | --- |
| CRQ | -- | 0.0007 | 0.0042 | 0.0031 | 0.0082 | 0.0099 | 0.0189 | 0.0059 | 0.0064 | 0.0056 | 0.0053 | 0.0079 | 0.0066 |
| MIS | (0.0001 - 0.0012) | -- | 0.0034 | 0.0031 | 0.0074 | 0.0086 | 0.018 | 0.0054 | 0.0058 | 0.0053 | 0.005 | 0.0088 | 0.0075 |
| SSI | (0.0033 - 0.0050) | (0.0026 - 0.0042) | -- | 0.0044 | 0.0099 | 0.0118 | 0.0201 | 0.0072 | 0.0079 | 0.0061 | 0.0067 | 0.0091 | 0.0092 |
| INK | (0.0023 - 0.0038) | (0.0024 - 0.0038) | (0.0036 - 0.0053) | -- | 0.0066 | 0.0083 | 0.0176 | 0.0066 | 0.0061 | 0.0057 | 0.0052 | 0.0095 | 0.0081 |
| TAB | (0.0073 - 0.0091) | (0.0065 - 0.0082) | (0.0089 - 0.0110) | (0.0057 - 0.0074) | -- | 0.0041 | 0.0162 | 0.0084 | 0.0079 | 0.0086 | 0.0085 | 0.0133 | 0.013 |
| MIR | (0.0090 - 0.0109) | (0.0077 - 0.0096) | (0.0107 - 0.0129) | (0.0075 - 0.0093) | (0.0033 - 0.0048) | -- | 0.0178 | 0.0102 | 0.0098 | 0.0104 | 0.0117 | 0.0152 | 0.015 |
| RIC | (0.0175 - 0.0202) | (0.0167 - 0.0192) | (0.0187 - 0.0215) | (0.0163 - 0.0189) | (0.0150 - 0.0174) | (0.0166 - 0.0191) | -- | 0.0177 | 0.0175 | 0.0157 | 0.0169 | 0.0205 | 0.0228 |
| BOU | (0.0052 - 0.0067) | (0.0046 - 0.0062) | (0.0063 - 0.0082) | (0.0058 - 0.0074) | (0.0075 - 0.0093) | (0.0093 - 0.0113) | (0.0166 - 0.0190) | -- | 0.0034 | 0.0035 | 0.0044 | 0.0090 | 0.0078 |
| COC | (0.0055 - 0.0072) | (0.0050 - 0.0066) | (0.0069 - 0.0089) | (0.0053 - 0.0070) | (0.0070 - 0.0088) | (0.0089 - 0.0108) | (0.0163 - 0.0188) | (0.0027 - 0.0041) | -- | 0.0030 | 0.0047 | 0.0099 | 0.0079 |
| SHD | (0.0048 - 0.0063) | (0.0046 - 0.0060) | (0.0052 - 0.0069) | (0.0049 - 0.0065) | (0.0077 - 0.0095) | (0.0094 - 0.0114) | (0.0146 - 0.0169) | (0.0028 - 0.0043) | (0.0023 - 0.0037) | -- | 0.0035 | 0.0079 | 0.0081 |
| SHM | (0.0045 - 0.0061) | (0.0042 - 0.0057) | (0.0058 - 0.0077) | (0.0044 - 0.0060) | (0.0077 - 0.0094) | (0.0107 - 0.0127) | (0.0157 - 0.0181) | (0.0036 - 0.0052) | (0.0039 - 0.0055) | (0.0027 - 0.0042) | -- | 0.0070 | 0.0072 |
| CRB | (0.0070 - 0.0089) | (0.0078 - 0.0098) | (0.0081 - 0.0102) | (0.0085 - 0.0105) | (0.0122 - 0.0145) | (0.0140 - 0.0164) | (0.0192 - 0.0219) | (0.0080 - 0.0100) | (0.0089 - 0.0110) | (0.0069 - 0.0088) | (0.0061 - 0.0080) | -- | 0.0098 |
| MAL | (0.0058 - 0.0075) | (0.0066 - 0.0084) | (0.0082 - 0.0103) | (0.0072 - 0.0090) | (0.0119 - 0.0141) | (0.0138 - 0.0163) | (0.0214 - 0.0243) | (0.0069 - 0.0087) | (0.0070 - 0.0089) | (0.0072 - 0.0091) | (0.0062 - 0.0081) | (0.0088 - 0.0108) | -- |
|  |  |  |  |  |  |  |  |  |  |  |  |  |  |

**Table S4** BLAST matches from sequences containing SNPs identified as being putatively adaptive in oyster populations in the studied region and being non-synonymous mutations. SNPs are located on the eastern oyster genome, chromosomes (Chr) and RADSeq loci (ID). Protein IDs and names are derived from the eastern oyster protein sequences of the genome. E-values from the two sequence variants were averaged from all SNPs of a locus. Molecular functions and biological processes related to the identified proteins were retrieved from SWISS-PROT database (Bairoch & Apweiler 2000) using protein names.

|  |  |  |  | **SNP position** | |  | |  | |  |  |
| --- | --- | --- | --- | --- | --- | --- | --- | --- | --- | --- | --- |
| **Chr** | **ID** | **Detection method** | **Correlated variable** | **Locus** | **Genome** | **Protein name and ID** | **E-values** | | **Molecular functions** | | **Biological processes** |
| NC_035780.1 | 503 | Polygenic | Number of weeks with temperature between 14 and 18°C | 59 | 13640138 | Tyrosine-protein kinase JAK2-like*, XP_022293938.1 | 1.27x10^-19^, 2.10x10^-20^ | | Non-membrane spanning protein tyrosine kinase activity, ATP binding | | Intracellular signal transduction |
|  | 2247 | Polygenic | Minimum monthly turbidity (m^-1^) | 66 | 231442 | Sulfotransferase family cytosolic 1B member 1-like isoform X1, XP_022301465.1 | 2.20x10^-38^, 4.04x10^-39^ | | Sulfotransferase activity | | NA |
|  | 2784 | Polygenic | Number of weeks with temperature between 14 and 18°C | 5, 31 | 25467620, 25467646 | MAM and LDL-receptor class A domain-containing protein 1-like, XP_022288512.1 | 2.89x10^-19^, 1.26x10^-18^ | | Vascular endothelial growth factor-activated receptor activity, scavenger receptor activity, semaphorin receptor activity, calcium ion binding, chitin binding | | Angiogenesis, chitin metabolic process, axon guidance |
|  | 4021 | Polygenic | Number of weeks with temperature between 14 and 18°C | 59 | 30824380 | Cubilin-like isoform X1, XP_022295199.1 | 8.84x10^-37^, 4.30x10^-36^ | | Metalloendopeptidase activity, scavenger receptor activity, polysaccharide binding, | | Immune response, integral component of membrane |
|  | 4541 | Polygenic | Number of weeks with temperature between 6 and 10°C | 1 | 32941643 | Dermatopontin-like, XP_022307992.1 | 8.11x10^-21^, 1.42x10^-22^ | | NA | | NA |
|  |  |  |  |  |  |  |  | |  | |  |
| NC_035781.1 | 17675 | Polygenic, Bayescan | Mean surface temperature (°C) | 12 | 43148252 | Uncharacterized protein LOC111119837*, XP_022316064.1 | 1.49x10^-15^, 3.41x10^-16^ | | NA | | NA |
|  | 17752 | Polygenic | Minimum monthly turbidity (m^-1^) | 51, 60, 66, 69 | 43394809, 43394818, 43394824, 43394827 | Uncharacterized protein LOC111120325, XP_022316755.1 | 4.04x10^-5^, 1.12x10^-5^ | | NA | | NA |
|  |  |  |  |  |  |  |  | |  | |  |
| NC_035782.1 | 21325 | Polygenic | Minimum monthly turbidity (m^-1^) | 30 | 10599711 | Uncharacterized protein LOC111124647, XP_022323424.1 | 1.28x10^-38^, 4.19x10^-39^ | | NA | | NA |
|  |  |  |  |  |  |  |  | |  | |  |
|  | 25659 | Polygenic | Number of weeks with temperature between 10 and 14°C | 20, 61 | 37991913, 37991954 | Zinc finger protein 493-like*, XP_022327115.1 | 4.13x10^-13^, 1.76x10^-16^ | | Nucleic acid binding, zinc ion binding | | Regulation of transcription, DNA-templated |
|  | 26869 | Polygenic | Mean surface temperature (°C) | 19, 45, 47, 51 | 45681700, 45681726, 45681728, 45681732 | Heat shock 70 kDa protein 12B-like, XP_022324405.1 | 6.83x10^-25^, 1.53x10^-24^ | | ATP binding | | NA |
|  | 30444 | Polygenic | Number of weeks with temperature between 10 and 14°C | 64 | 61862631 | Uncharacterized protein LOC111122921*, XP_022320665.1 | 3.41x10^-15^, 5.67x10^-15^ | | NA | | NA |
|  | 30445 | Polygenic | Number of weeks with temperature between 10 and 14°C | 25 | 61862761 | Uncharacterized protein LOC111122921*, XP_022320665.1 | 1.11x10^-13^, 1.08x10^-13^ | | NA | | NA |
|  |  |  |  |  |  |  |  | |  | |  |
| NC_035783.1 | 34155 | Polygenic | Number of weeks with temperature between 10 and 14°C | 63 | 14599961 | Organic cation transporter protein-like, XP_022332356.1 | 2.73x10^-39^, 1.19x10^-38^ | | Aconitate hydratase activity, 2-methylisocitrate dehydratase activity, metallocarboxypeptidase activity, organic anion transmembrane transporter activity, mRNA 3'-UTR binding, zinc ion binding, 4 iron, 4 sulfur cluster binding | | Carboxylic acid metabolic process, cellular metabolic process, tricarboxylic acid cycle, propionate metabolic process, methylcitrate cycle, regulation of sporulation, transmembrane transport, integral component of membrane |
|  | 37497 | Polygenic | Number of weeks with temperature between 10 and 14°C | 44, 74 | 29953167, 29953197 | DBH-like monooxygenase protein 1*, XP_022328761.1 | 1.19x10^-24^, 1.24x10^-25^ | | Protein kinase activity, dopamine beta-monooxygenase activity, tyramine-beta hydroxylase activity, G-protein coupled receptor activity, ATP binding, SNAP receptor activity, ubiquitin protein ligase binding, copper ion binding, nucleic acid binding | | Ubiquitin-dependent protein catabolic process, cellular nitrogen compound metabolic process, octopamine biosynthetic process, dopamine catabolic process, norepinephrine biosynthetic process, viral life cycle, intracellular protein transport, vesicle-mediated transport |
|  |  |  |  |  |  |  |  | |  | |  |
| NC_035784.1 | 47432 | Polygenic | Number of weeks with temperature above 18°C | 12, 33, 84 | 2944535, 2944586, 2944607 | Lens fiber membrane intrinsic protein-like, XP_022338988.1 | 4.47x10^-32^, 4.40x10^-33^ | | Structural constituent of eye lens, water channel activity, protein dimerization activity | | Protein tetramerization, homophilic cell adhesion via plasma membrane adhesion molecules, heterophilic cell-cell adhesion via plasma membrane cell adhesion molecules |
|  | 58337 | Polygenic | Minimum monthly turbidity (m^-1^) | 3 | 81976217 | Monocarboxylate transporter 12-like isoform X2, XP_022339928.1 | 1.95x10^-39^, 6.11x10^-39^ | | NA | | Transmembrane transport |
|  |  |  |  |  |  |  |  | |  | |  |
| NC_035785.1 | 64635 | Polygenic | Minimum surface temperature (°C) | 16 | 3421293 | Sushi, nidogen and EGF-like domain-containing protein 1 isoform X1, XP_022286589.1 | 3.55x10^-16^, 3.11x10^-16^ | | Calcium ion binding, cell-matrix adhesion | | NA |
|  |  |  |  |  |  |  |  | |  | |  |
| NC_035787.1 | 76833 | Polygenic | Number of weeks with temperature between 14 and 18°C | 16, 71, 84 | 28740756, 28740769, 28740824 | Cytochrome P450 2D9-like*, XP_022298084.1 | 3.11x10^-25^, 4.50x10^-23^ | | Iron ion binding, heme binding, monooxygenase activity, oxidoreductase activity, acting on paired donors, with incorporation or reduction of molecular oxygen | | NA |
|  | 129926 | Polygenic | Minimum surface temperature (°C) | 52 | 4860696 | Uncharacterized protein LOC111107835, XP_022298929.1 | 9.31x10^-26^, 5.60x10^-27^ | | NA | | NA |
|  |  |  |  |  |  |  |  | |  | |  |
| NC_035788.1 | 95399 | Polygenic | Number of weeks with temperature between 6 and 10°C | 22, 29, 70 | 81061379, 81061386, 81061427 | Glycoprotein 3-alpha-L-fucosyltransferase A-like isoform X3, | 9.14x10^-44^, 2.46x10^-43^ | | NA | | NA |
|  |  |  |  |  |  |  |  | |  | |  |
| NC_035789.1 | 97546 | Polygenic | Mean surface temperature (°C) | 87 | 11514741 | Receptor-type tyrosine-protein phosphatase epsilon-like isoform X5, XP_022311522.1 | 2.84x10^-40^, 1.34x10^-39^ | | Protein tyrosine phosphatase activity | | NA |
|  |  |  |  |  |  |  |  | |  | |  |

*Protein sequence identified as ‘Low quality protein’ in SWISS-PROT.

**References**

Bairoch, A., & Apweiler, R. (2000). The SWISS-PROT protein sequence database and its supplement TREMBL in 2000. *Nucleic Acids Research*, 28, 45–48.

**Fig. S1** Position of SNPs within the 10 eastern oyster chromosomes. SNPs are represented by horizontal lines (total = 11321 SNPs).


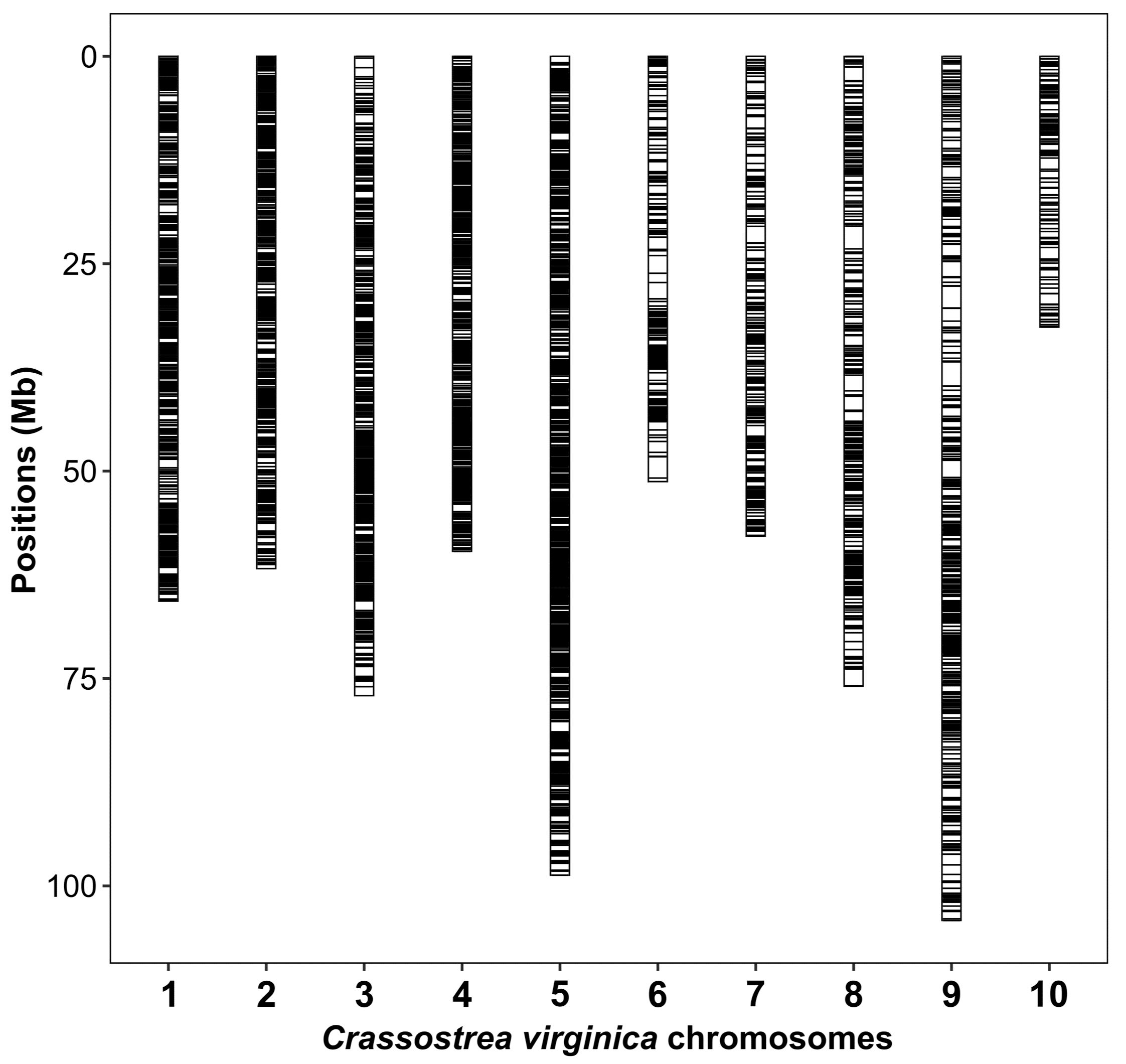


**Fig. S2** Result of the principal component analysis (PCA) of missing data (11 321 SNPs) by sequencing lane. a) Values for PC1 and PC2; b) Values for PC1 and PC3; c) Values for PC1 and PC4; d) Values for PC2 and PC3; e) Values for PC3 and PC4; f) Values for PC3 and PC4.


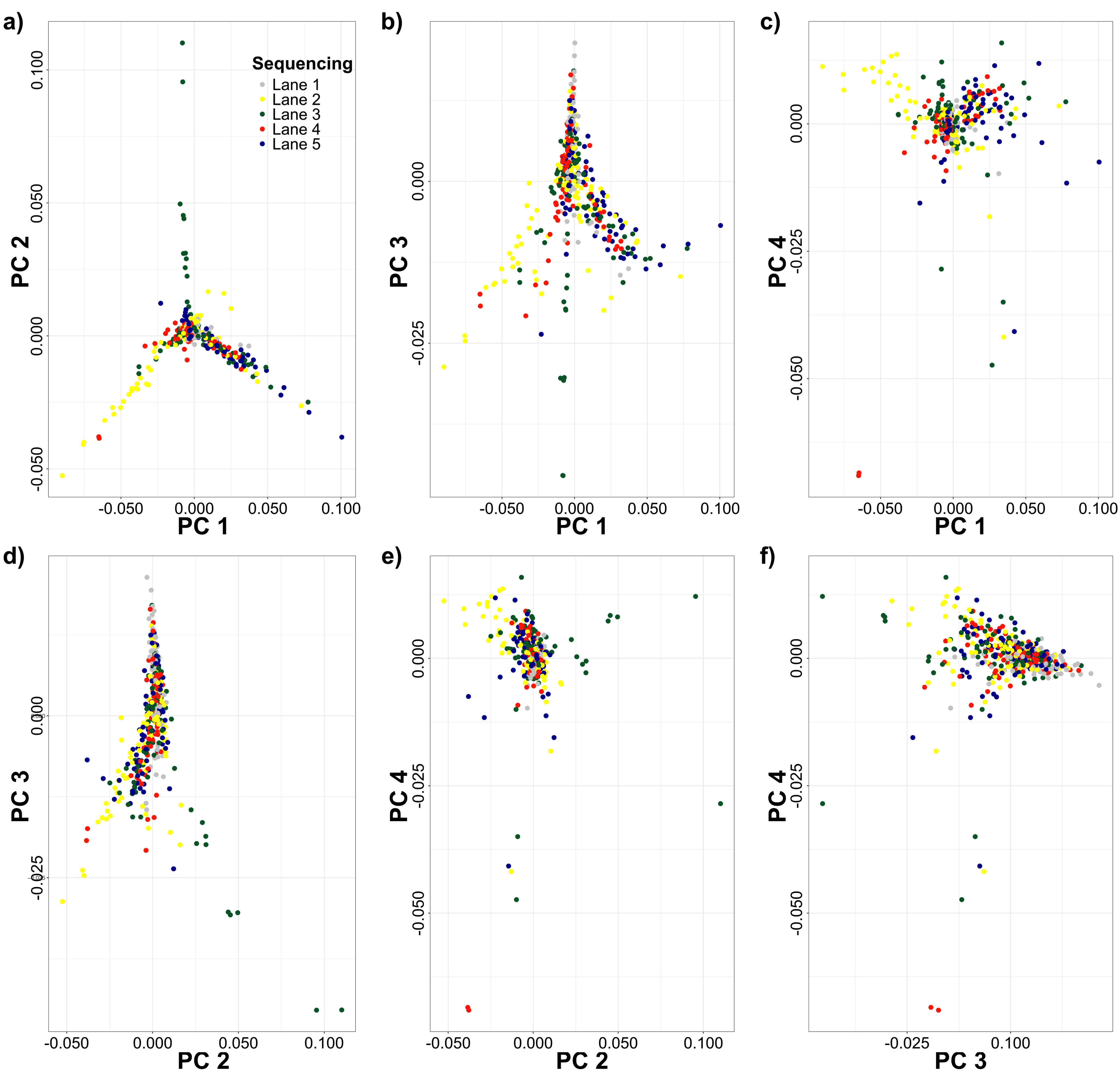


**Fig. S3** Result of the principal component analysis of missing data (11 321 SNPs) by sampling site. a) Values for PC1 and PC2; b) Values for PC1 and PC3; c) Values for PC1 and PC4; d) Values for PC2 and PC3; e) Values for PC3 and PC4; f) Values for PC3 and PC4.


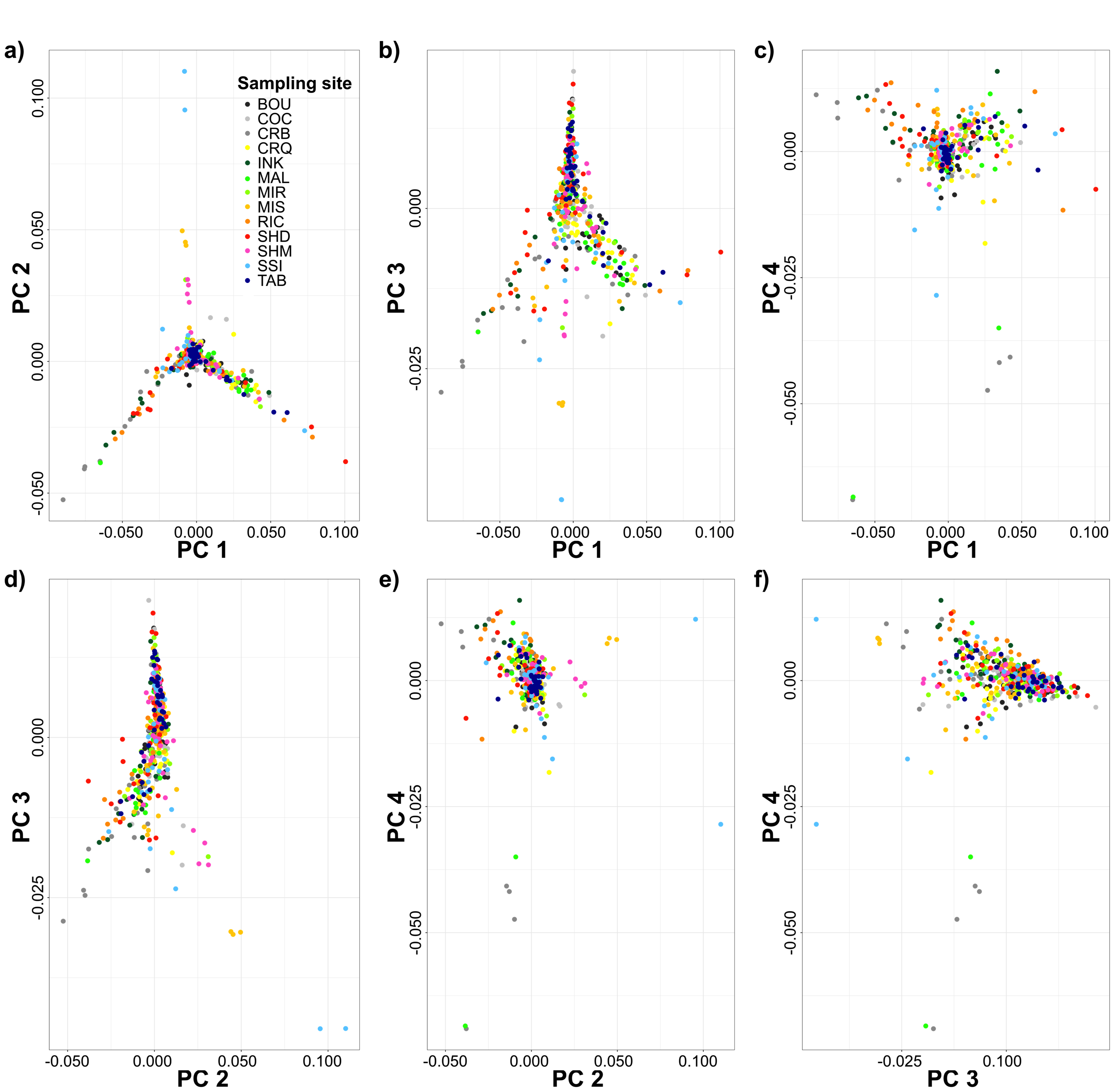


**Fig. S4** Isolation-by-distance (IBD) relationship between all sampling sites pairs, where linearized pairwise *F*_ST_ values (*F*_ST_/(1- *F*_ST_)) are regressed over marine distance. Circles represent pairwise comparisons and the regression line (blue) is fitted with a 95% confidence limits (grey).


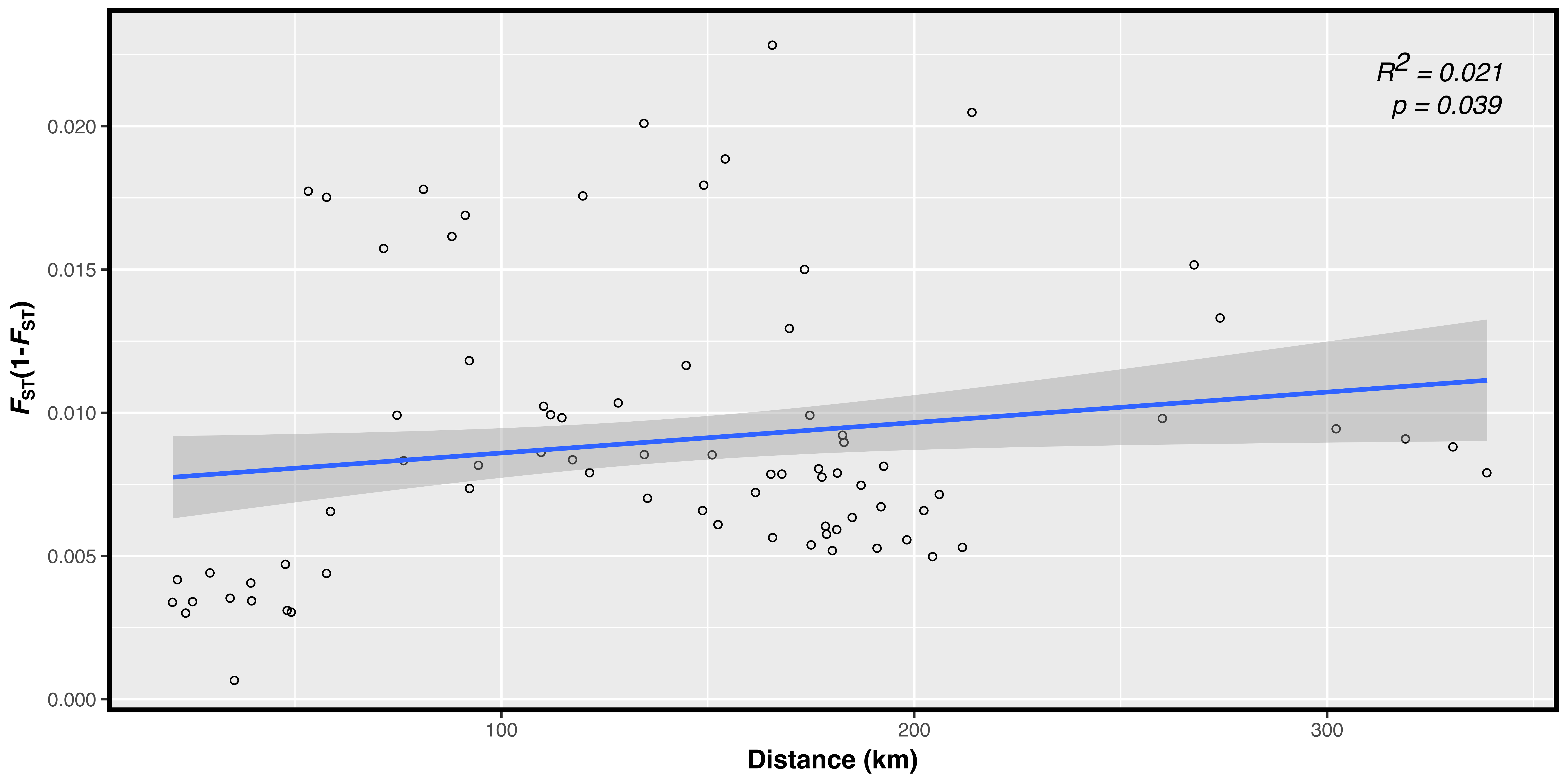


**Fig. S5** Bayesian information criterion (BIC) values versus number of clusters (K = 1 to 14) for the discriminant analysis of principal components (DAPC) using 8 246 neutral SNPs.


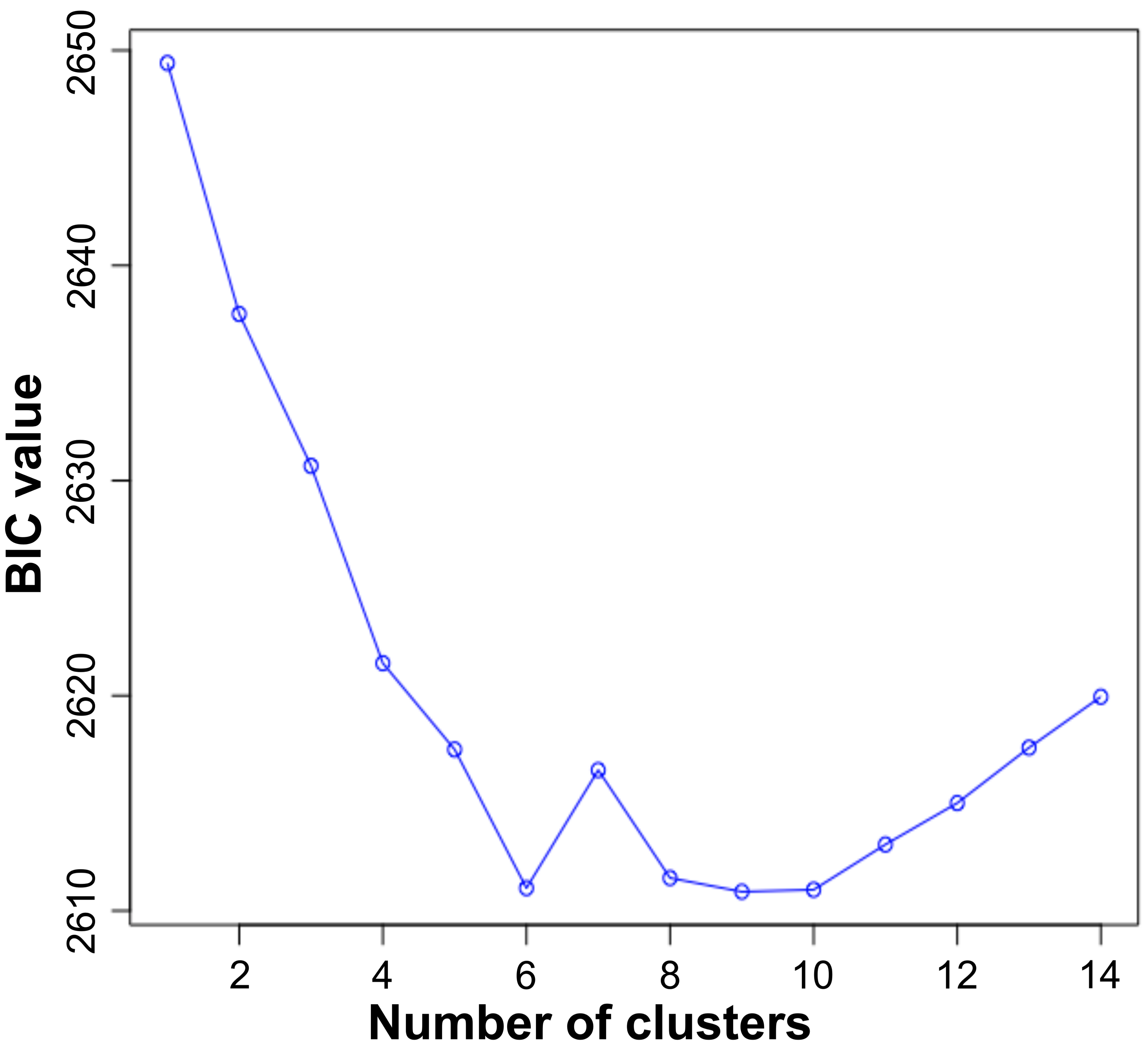


**Fig. S6** Discriminant analysis of principal components (DAPC) of genetic differentiation among sampling sites based on 8 246 neutral SNPs. a) Values for DPC1 and DPC2; b) Values for DPC3 and DPC4; c) Values for DPC5 and DPC6. Individuals are represented as dots, each color represents a unique sampling site and 67% inertia ellipse is presented for each site.


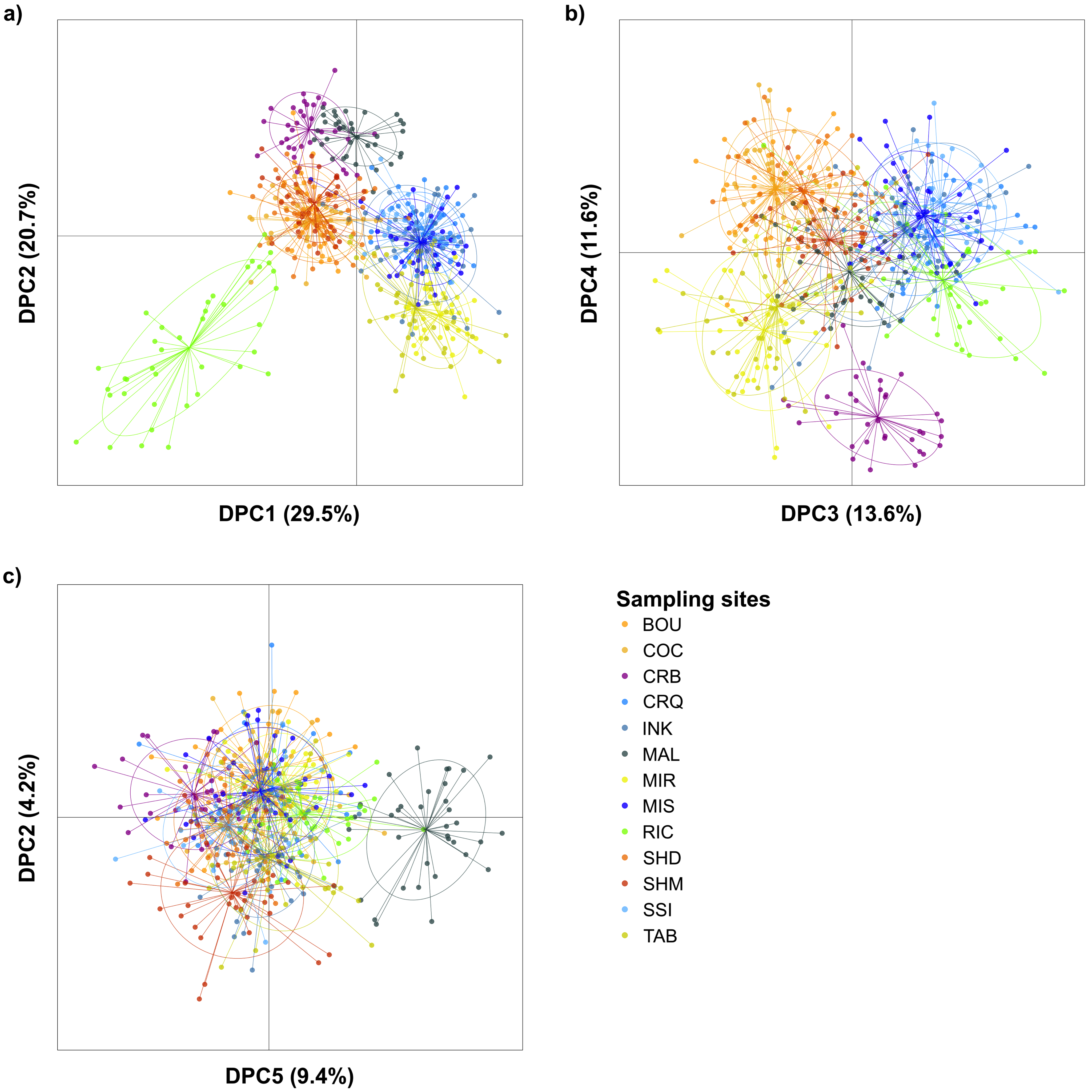


**Fig. S7** Assignment of individual oysters to their original cluster (grey circles) or another cluster (white circles) using all samples (a) and equal sample size (*n* =33). Numbers in circles represent the percentage of individuals from a cluster assigned to another. Circles diameter is proportional to percentage. Numbers in parenthesis represent the percentage of individuals from a ‘current cluster’ tagged as migrants from a ‘inferred cluster’.


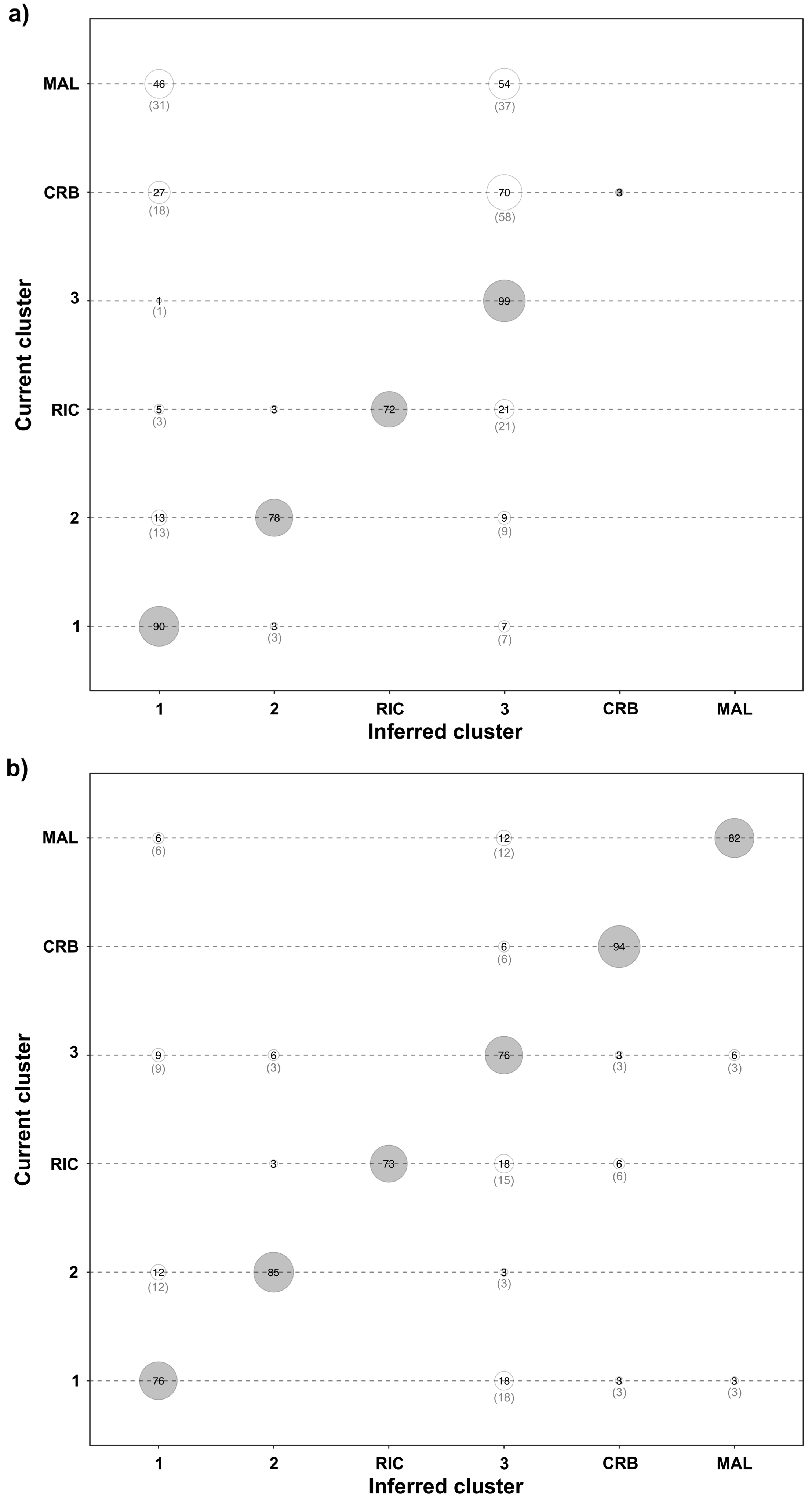


**Fig. S8** Non-significant correlations between additive individual polygenic score based on (a) minimum monthly turbidity and 23 SNPs, (b) maximum monthly turbidity and 8 SNPs. Correlation coefficient (R^2^) and *P*-values of the linear (a) or quadratic (b) models are presented for each variable. For minimum monthly turbidity, a single outlier polygenic score was removed after visualizing the results, the correlation was recalculated and became non-significant.


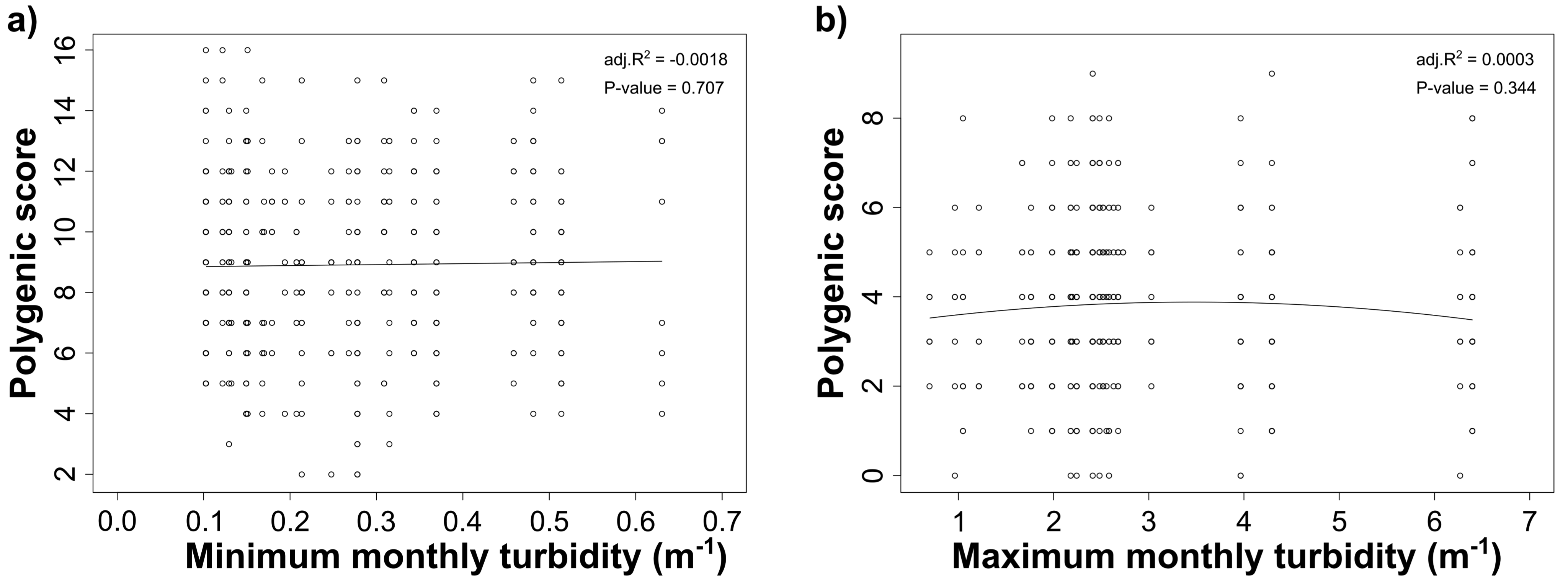

Supplement: Supplementary file 1 [file EVA-12-587-s001.docx]
